# An intercomparison study of ELISAs for the detection of porcine reproductive and respiratory syndrome virus – evaluating six conditionally dependent tests

**DOI:** 10.1371/journal.pone.0262944

**Published:** 2022-01-25

**Authors:** Clara Schoneberg, Jens Böttcher, Britta Janowetz, Anja Rostalski, Lothar Kreienbrock, Amely Campe

**Affiliations:** 1 Department of Biometry, Epidemiology and Information Processing, WHO Collaborating Centre for Research and Training for Health in the Human-Animal-Environment Interface, University for Veterinary Medicine Hannover, Hannover, Germany; 2 Bavarian Animal Health Service, Poing, Germany; Sciensano, BELGIUM

## Abstract

Latent class analysis is a widely used statistical method for evaluating diagnostic tests without any gold standard. It requires the results of at least two tests applied to the same individuals. Based on the resulting response patterns, the method estimates the test accuracy and the unknown disease status for all individuals in the sample. An important assumption is the conditional independence of the tests. If tests with the same biological principle are used, the assumption is not fulfilled, which may lead to biased results. In a recent publication, we developed a method that considers the dependencies in the latent class model and estimates all parameters using frequentist methods. Here, we evaluate the practicability of the method by applying it to the results of six ELISA tests for antibodies against the porcine reproductive and respiratory syndrome (PRRS) virus in pigs that generally follow the same biological principle. First, we present different methods of identifying suitable starting values for the algorithm and apply these to the dataset and a vaccinated subgroup. We present the calculated values of the test accuracies, the estimated proportion of antibody-positive animals and the dependency structure for both datasets. Different starting values led to matching results for the entire dataset. For the vaccinated subgroup, the results were more dependent on the selected starting values. All six ELISA tests are well suited to detect antibodies against PRRS virus, whereas none of the tests had the best values for sensitivity and specificity simultaneously. The results thus show that the method used is able to determine the parameter values of conditionally dependent tests with suitable starting values. The choice of test should be based on the general fit-for-purpose concept and the population under study.

## Introduction

Porcine reproductive and respiratory syndrome (PRRS) is a disease in pigs caused by the *Betaarterivirus suid* (PRRSV). Two genotypes (1 and 2) are generally distinguished. Genotype 1 originated from Europe, and genotype 2 originated from North America; viruses within genotypes are not antigenically homogenous [1]. Infection is associated with late-term abortion in sows and respiratory disease in weaned and fattening pigs and thus results in significant economic losses worldwide [2]. Vaccination is frequently practiced to prevent clinical disease; currently, one genotype 2 and four genotype 1 live attenuated vaccines are commercially available in Germany.

After the discovery of the viral etiology of PRRS in the early nineties of the last century, an immune peroxidase monolayer assay (IPMA) was developed to detect antibodies [3]. Viral propagation in cell culture was extremely difficult. Initially, PRRSV was multiplied in primary lung alveolar macrophages, and later, the virus adapted to a permanent cell line (MARC145) [4]. Although the neutralization test is frequently regarded as a reliable gold standard for the detection of antibodies against viruses, in the case of PRRSV, neutralizing antibodies are developed only late in the course of infection and are quite specific to viral subtypes within genotypes [5, 6]. In contrast, IPMA allowed the detection of cross-reactive antibodies directed to the conserved and abundantly present nucleocapsid antigen. Finally, as IPMA was difficult to standardize, a patent-protected ELISA was developed and commercialized (see Test 1). Since then, a huge body of experience has accumulated, and this ELISA has been established in worldwide laboratories. After the patent expired, additional ELISAs were developed and required validation. The question for a proper gold standard thus re-emerged. At least for some authors, it became a matter of course to regard the existing ELISA as a gold standard [7]. However, this view precludes any further improvement of antibody ELISAs; therefore, an alternative approach is required.

In a voluntary study by the Bavarian Animal Health Service, six of these ELISAs were used on sows, gilts and piglets from farms in southeast Germany with different vaccination statuses. The aim of the study was to estimate the accuracy of the tests and to assess the applicability of the newly developed ELISAs both under in-field conditions.

Generally, diagnostic test evaluation is performed by comparison to a gold standard test [8]. If there is no gold standard or if it only has suboptimal accuracies, a comparison may lead to incorrect values [9]. The application of the tests to a mostly small group of animals with a known disease status leads to results that only apply to the observed population. Therefore, latent class analysis (LCA) is often used as a method for estimating the diagnostic test accuracy for individuals with unknown disease status without a gold standard [10].

The LCA assumes that an underlying latent structure exists and that the diagnostic tests that measure the true unknown disease status of the individuals represent imperfect indicator variables under investigation. Based on the response patterns of the tests, both the accuracy of the tests and the proportion of both disease statuses (positive/negative) are discovered by the model. Hence, the test performance under the given conditions, such as the structure of the population in terms of age, gender, race, immune status, etc., can be estimated. The parameters may be estimated using Bayesian [11] or frequentist methods [12, 13].

A requirement of LCA is the conditional independence of the diagnostic tests [14]. This means that within a latent class, the result of one test does not allow conclusions to be drawn about the results of the other tests. However, if infectious diseases are considered, this condition is often not met. Conditionally independent tests use different biological principles (e.g., detection of antigen or antibodies), which cannot always be detected at the same time of infection [15]. Thus, these tests do not measure the same latent status, and applying the LCA leads to biased results. Therefore, LCA requires tests with the same biological basis, which means that they are conditionally dependent. This is the case in the study for the detection of PRRSV. All tests used assess the presence of antibodies against the virus, i.e., they measure the same latent status.

There are already some approaches that consider conditional dependencies both in the Bayesian [16–18] and frequentist [19–24] frameworks. These methods often require some assumptions, such as a large number of diagnostic tests or accurate prior distributions. We developed a frequentist latent class approach, which takes the dependencies of the tests into account, thus no longer requiring the assumption of conditional independence [25]. In addition, this method is easily applicable in many situations even when little prior information is accessible. This approach expands the LCA by a term for the dependency and estimates the parameters alternately in an iterative process until convergence occurs. We showed in simulation studies that this method is better than the classic conditionally independent LCA in many situations and provides results similar to the Bayesian approach for conditionally dependent tests; however, in contrast this approach does not need any prior distribution.

In this publication, we show that our iterative, frequentist latent class approach is applicable to real-world data by evaluating the results of the mentioned voluntary study by the Bavarian Animal Health Service containing the results of six different ELISA tests against the PRRS virus for pigs in southern Germany. We present the results of the analysis of the entire dataset as well as a vaccinated subgroup of animals. Different approaches for the starting values of the algorithm are considered, and the resulting parameter values are compared.

## Material and methods

### Sample collection and diagnostics applied

Between 2016 and 2018, the Bavarian Animal Health Service offered voluntary PRRSV immune control to pig farmers in southeast Germany. Therefore, per farm, blood samples were collected from ten weaned piglets at 8–12 weeks, six gilts and 18 sows of different parities (1./2., 3./4. and 5./6. parity). After initial testing (PCR, ELISA Test 1, neutralization tests against and IFN-recall assay after stimulation of whole blood with a panel of PRRSV-vaccine virus strains), sera were stored at -20°C. The current vaccination status of the herd was assessed by a face-to-face questionnaire. Twenty-four farms in 13 districts participated. In 2019, sera were tested for antibodies against the virus by six different ELISA tests from five manufacturers:

- PRRS X3, Idexx (Test 1)
- pigtyp PRRSV Ab, Indical (Test 2)
- ID Screen PRRS indirect, IDVet (Test 3)
- Ingezim PRRS 2.0, Ingenasa (Test 4)
- Ingezim PRRS universal, Ingenasa (Test 5)
- PrioCHECK, ThermoFisher (Test 6).

A single batch was used for each ELISA, and all sera per test were analyzed in one run. The farms had been immunized in different ways in the past. Fourteen farms received vaccinations against genotype 1 of the virus, 4 farms received vaccinations against genotype 2 of the virus, 1 farm was vaccinated against both genotypes, and 5 farms did not have any vaccination in the past. To ensure comparability of the test results, only tests that checked for both genotypes of the virus were used.

In this analysis data from 812 pigs were evaluated. Overall, 8 animals with incomplete results were documented; therefore, they were removed from further analysis. Thus, 804 animals were included in the statistical evaluation.

The analysis of the dataset determined the test accuracies and the prevalence in a sample selected from the entire population without specific selection criteria. The sample consisted of animals with different vaccination statuses: unvaccinated animals and animals vaccinated against genotype 1, genotype 2 or both genotypes of the virus. Thus, great heterogeneity in the immune status of the animals is present as a typical sample from pig farms in Germany. To examine whether this heterogeneity impacts the performance of the method and how the test accuracy and prevalence change, we applied the methods used to a uniformly vaccinated population for an additional analysis. To examine a group as homogeneous as possible with a large number of cases, we considered the 468 samples on the 14 farms vaccinated against European genotype 1 of the virus for the second part of the analysis.

### The latent class model

The intercomparison of diagnostic tests was performed with the method introduced recently using a modified latent class model [25]. In a latent class analysis, it is assumed that there is a latent variable with two classes. When applied to diagnostic tests, these classes (diseased vs. not diseased) are defined by the observed diagnostic tests, which assume two values (positive/negative). Each individual tested by all diagnostic tests analyzed in the LCA represents an observation in the model. Thus, the proportion of the positive class, i.e., the prevalence, and the response probability of each test in these classes (i.e., the test accuracies) can be determined for the sample. Our model also considers conditional dependencies between the tests due to similar test principles by integrating an additional dependency term into the model.

Formally, in the modified model, it is assumed that there is a latent variable with two classes, which is measured by *M* observed variables. Observation *Y*_*i*_ = (*Y*_*i*1_,…,*Y*_*iM*_), *i* = 1, …, *N* represents individual i’s response pattern with the possible values *r*_*m*_ = 0,1 for observation *Y*_*im*_. The probability of membership in latent class *c* can be expressed as *γ*_*c*_ with $\sum_{c=0}^{1}\gamma_{c}=1$, and the probability of the response *r*_*m*_ to variable *m* in class *c* can be expressed as $\rho_{m,r_{m}|c}$. Let *I*(·) be the indicator function. Then, the likelihood of parameters *γ* and *ρ* for the observations Y can be written in the following form:

$$L\left(\gamma ,\rho |Y\right)=\sum_{i=1}^{N}\sum_{c=0}^{1}\gamma_{c}\left(\prod_{m=1}^{M}\prod_{r_{m}=0}^{1}\rho_{m,r_{m}|c}^{I\left(Y_{im}=r_{m}\right)}+\eta_{r_{1},\ldots ,r_{M}}^{c}\right),$$ (a)

where $\eta_{r_{1},\ldots ,r_{M}}^{c}$ is a term for the influence of the $\sum_{i=2}^{M}\left(\begin{array}{c} M\\ i \end{array}\right)$ conditional two-way to *M*-way dependencies of all *M* tests in class *c* on the likelihood of the respective observed response patterns as described in [25]. The conditional dependencies are expressed in terms of the conditional covariances between the test results in the two latent classes, respectively. They are considered as the differences between the observed proportion of matching correct responses of two to M tests in latent class *c* and the expected corresponding proportion in class *c* under the conditional independence assumption.

All parameter values from Formula (a) are estimated in an iterative algorithm:

1. Appropriate starting values are chosen for the prevalence, test accuracies and dependencies.
2. The dependencies are considered fixed. The test accuracies and the prevalence are newly estimated by an expectation maximization (EM) algorithm in the same manner as noted in the conditionally independent LCA (such as in [13]).
3. Using the test accuracies and the prevalence estimated in the previous step, the expected frequencies in the case of conditional independence are redetermined. The dependencies can then be recalculated from the difference to the observed frequencies.
4. The algorithm starts again with step 2 until it converges, i.e., the difference of the log-likelihood of two successive steps falls below a specified limit or the algorithm reaches the maximum number of 1000 iterations.

Confidence intervals for test accuracies and the prevalence are calculated using the normal approximation interval for binomial distributions. The model is applicable if the results of at least three tests, each with two possible answers (diseased / not diseased), are available. The procedure was implemented in R (version 3.5.0; [26]); see S1 File.

### Starting values

In a previous investigation, we showed that the applied iterative frequentist approach of the LCA needs well-chosen starting values to converge to the true underlying values [25]. Therefore, we used three sources of information for the parameters required: the manufacturers’ evaluation studies conducted for every commercially available test, previous publications evaluating the diagnostic tests used in this study [7, 27–34] and experience from application from the researchers of the Bavarian Animal Health Service. Additionally, we used the examined dataset to estimate the prevalence in the sample.

**Fig 1:**
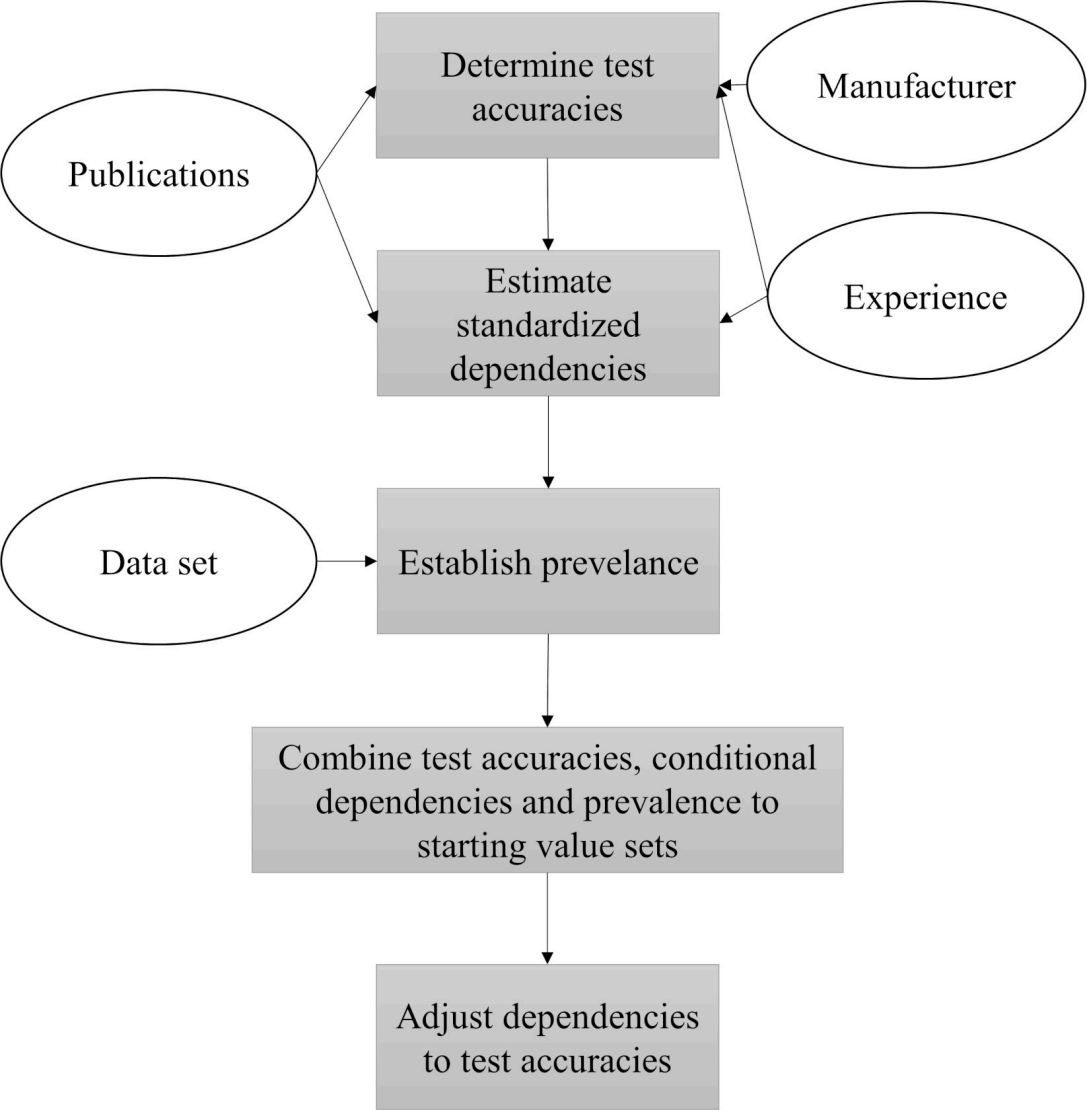
Step-by-step procedure for determining the starting values when taking various information sources into account.

Based on these sources, we proceeded as follows to determine the starting values that can be found in S1 and S3 Tables (see Fig 1 also):

1. Determine test accuracies based on each available source:
  - Adopt values from the manufacturer’s evaluation studies (indicated as M).
  - Find previous publications (indicated as P) evaluating the diagnostic tests used in this study (see S5 Table) and calculate a weighted average by considering the studies that have a similar structure and study population more strongly.
  - Ask researchers (indicated as R) from the associated laboratories (here, the Bavarian Animal Health Service) for an assessment of the test accuracies based on their experience in application and the values of the other sources.
2. Estimate the dependencies of the tests in terms of their correlation (as described in [25]) based on each available source, so that these values are standardized and independent of the test accuracies:
  - Find previous publications evaluating the diagnostic tests used in this study and use their values for conditional dependency (here, we could not find any previous publications considering a dependency of the tests, so this source did not provide any information on the conditional dependency of the tests).
  - Ask researchers (indicated as R) from the associated laboratories (here, the Bavarian Animal Health Service) for an assessment of the dependencies of the tests used based on their experience and the biological principles of the tests used.
3. Establish prevalence for the dataset under consideration by estimating the approximate proportion of positive tests in the dataset, considering the chosen starting values of the test accuracies from step 1 (here, using the dataset (Table 1) with a slightly higher prevalence, as all sources in step 1 assume a higher sensitivity than specificity for all six tests).
4. Define starting value datasets: All available information on the test accuracies, the dependencies and the prevalence are combined. Every possible combination forms a starting value set. The exact number of the resulting sets depends on the amount of prior information. At least four different starting values should be applied so that two different dependency structures and two different test accuracy sets are used to examine the stability of the results. If there is only one source for the dependencies, independence of the tests (indicated as I) can also be used as a starting value to test stability of the method.
  Here, that means all three starting values for the test accuracies (M, P, R) are combined with the information for prevalence (step 3) and conditional dependencies (R). In addition, all three values for the test accuracies are used as starting values with the assumption of conditional independence (I). This leads to six different starting value sets (MI, MR, PI, PR, RI, RR, see S1 Table).
5. Adjust the standardized values of the dependencies to the selected test accuracies M, P, R and the prevalence (step 3) so that the conditional dependencies are described in terms of conditional covariances and any combination of the test results has a positive probability (see [25]). This leads to deviating values of the conditional covariances depending on the selected starting values for the test accuracies.

**Table 1:** Observed frequencies of the two results for all six tests used in the latent class analysis of the complete dataset (percentage rounded to one digit).

	Positive tested Animals (in %)	Negative tested Animals (in %)
Test 1	549 (68.3)	255 (31.7)
Test 2	518 (64.4)	286 (35.6)
Test 3	487 (60.6)	317 (39.4)
Test 4	461 (57.3)	343 (42.7)
Test 5	578 (71.9)	226 (28.1)
Test 6	623 (77.5)	181 (22.5)

The above-described steps for the starting values 1a and 1c were performed a second time for the vaccinated subgroup. Since this is a subgroup of the first dataset, we assumed that it has parameters similar to those of the entire dataset. Therefore, we selected the starting values of the test accuracies that differ the most from one another to assess the stability of the model. All starting values for the vaccinated subgroup can be found in S2 Table.

## Results

In the following section, we present three main results of the analysis. First, we show the parameter values calculated by the method for the complete dataset. Second, these values are considered in context with the selected starting values. Finally, the results are compared with those of the vaccinated subgroup to obtain an impression of the influence a vaccination has on the performance of the method proposed and the resulting parameter values. The starting value sets MI, MR, PI, PR, RI, RR specified in the last chapter are used in the calculation, where M indicates the information provided by the manufacturer, R denotes the information on test accuracies and dependencies provided by the researchers, P indicates the values from previous publications and I represents the assumptions of independent tests.

### Overall results and effect of starting values

In general, all calculations agreed that slightly greater than three-quarters (75.3–76.8%) of the animals in the sample examined were assigned to the positive latent class, i.e., had antibodies against the virus. Test 6 (PrioCHECK) had the highest estimated sensitivity values, and test 4 (Ingezim PRRS 2.0) had the lowest estimated sensitivity values (see Table 2). The values of the remaining tests were distributed evenly between these results. Overall, the specificities had higher values, which were between 95% and 99% for tests 1 to 4. Tests 5 (Ingezim PRRS universal) and 6 had lower specificities of approximately 88% and 78%, respectively. Test 6 in particular produced an extended number of false-positive results. Test 1 (PRRS X3) had the highest values of sensitivity and specificity combined with minimal differences compared with the other tests considered. These results were consistent with the observed frequencies (Table 1).

**Table 2:** Estimated values for the prevalence and the test accuracies for the six starting value sets for the complete sample with confidence limits reported in brackets (rounded to one digit).

Parameters estimated	Starting value sets					
	Set MI	Set MR	Set PI	Set PR	Set RI	Set RR
Prevalence	75.5	76.7	75.7	76.8	75.3	76.4
in %	[72.5, 78.4]	[73.7, 79.6]	[72.7, 78,6]	[73.9, 79.7]	[72.3, 78.3]	[73.4, 79.3]
Sensitivity in %						
Test 1	88.9	87.7	88.6	87.6	89.0	87.9
	[86.7, 91.0]	[85.4, 89.9]	[86.5, 90.8]	[85.3, 89.8]	[86.8, 91.2]	[85.7, 90.2]
Test 2	84.4	83.1	84.2	83.0	84.5	83.4
	[81.9, 86.9]	[80.5, 85.7]	[81.6, 86.7]	[80.4, 85.6]	[82.0, 87.0]	[80.8, 85.9]
Test 3	79.7	78.5	79.5	78.3	79.8	78.7
	[76.9, 82.5]	[75.6, 81.3]	[76.7, 82.3]	[75.5, 81.2]	[77.1, 82.6]	[75.9, 81.5]
Test 4	75.6	74.5	75.4	74.4	75.8	74.8
	[72.6, 78.6]	[71.5, 77.5]	[72.4, 78.4]	[71.4, 77.4]	[72.8, 78.7]	[71.8, 77.8]
Test 5	91.3	90.5	91.1	90.4	91.4	90.7
	[89.3, 93.2]	[88.5, 92.5]	[89.2, 93.1]	[88.3, 92.4]	[89.5, 93.4]	[88.7, 92.7]
Test 6	95.3	95.0	95.1	94.9	95.3	95.1
	[93.8, 96.7]	[93.5, 96.5]	[93.7, 96.6]	[93.4, 96.4]	[93.9, 96.8]	[93.7, 96.6]
Specificity in %						
Test 1	94.9	95.4	95.1	95.5	94.8	95.2
	[93.4, 96.4]	[94.0, 96.9]	[93.6, 96.6]	[94.1, 97.0]	[93.3, 96.4]	[93.8, 96.7]
Test 2	96.8	96.9	96.9	96.9	96.8	96.8
	[95.6, 98.1]	[95.7, 98.1]	[95.7, 98.1]	[95.8, 98.1]	[95.6, 98.0]	[95.5, 98.0]
Test 3	98.2	98.2	98.3	98.2	98.2	98.1
	[97.3, 99.1]	[97.2, 99.1]	[97.4, 99.2]	[97.3, 99.1]	[97.2, 99.1]	[97.2, 99.0]
Test 4	98.8	99.1	98.9	99.1	98.8	99.0
	[98.1, 99.6]	[98.4, 99.7]	[98.1, 99.6]	[98.4, 99.7]	[98.1, 99.6]	[98.3, 99.7]
Test 5	87.8	89.2	88.0	89.2	87.6	88.9
	[85.5, 90.0]	[87.0, 91.3]	[85.7, 90.2]	[87.1, 91.4]	[85.3, 89.9]	[86.8, 91.1]
Test 6	77.1	80.0	77.4	80.1	77.0	79.6
	[74.2, 80.0]	[77.2, 82.7]	[74.5, 80.3]	[77.4, 82.9]	[74.0, 80.0]	[76.8, 82.4]

In the negative latent class, tests 1, 2, 3 and 4 had strong pairwise dependencies. Tests 5 and 6 were less dependent on each other and on the other tests. This yields a greater proportion of the agreeing negative results in the first four tests due to simultaneous incorrect results than in the other two tests. All pairwise dependencies of the tests were substantially higher within the positive latent class. Tests 1, 5 and 6 had the highest dependencies in the positive latent class, which were almost twice as large as the strongest dependencies in the negative class. Therefore, the dependency structures in the two classes differ both in the tests with the highest dependencies and in the size of these values.

The calculated dependencies of the tests for all six sets of starting values can be found in S3 Table.

All six starting value sets led to very similar results in the test accuracies and the prevalence (Table 2). The estimates of prevalence and sensitivity exhibited maximum deviations of 1.5%. The results for the specificity had an even greater consistency. Only the estimations of the specificity of test 6 varied by up to 3%. Thus, all six runs of the algorithm yielded consistent results despite differing starting values in contrast to the first simulation study [25].

### Vaccinated subgroup

As the evaluation of diagnostic tests is related to the population under study, we additionally compared the results with the subgroup of farms that had participated in a vaccination programme before our investigation. The results in this vaccinated subgroup were less clear. Starting value set 1 led to a significantly reduced prevalence and lower values for all specificities (see Table 3). According to these results, the vaccinated subgroup included 68.7% antibody-positive animals. The specificities were between 89.8% for test 4 and 41.5% for test 6. On the other hand, the sensitivity was (almost) 100% for all six tests.

**Table 3:** Resulting values for the prevalence and the test accuracies for starting value set RI (test accuracies estimated by researchers with assumption of independent tests) for the complete sample and the vaccinated subgroup with confidence limits reported in brackets (rounded to one digit).

Starting value sets	Complete dataset	Vaccinated subgroup
Prevalence in %	75.3 [72.3, 78.3]	68.7 [64.6, 72.9]
Sensitivity in %		
Test 1	89.0 [86.8, 91.2]	100.0 [99.7, 100.0]
Test 2	84.5 [82.0, 87.0]	98.2 [97.0, 99.4]
Test 3	79.8 [77.1, 82.6]	97.4 [95.9, 98.8]
Test 4	75.8 [72.8, 78.7]	95.5 [93.6, 97.3]
Test 5	91.4 [89.5, 93.4]	100.0 [100.0, 0.0]
Test 6	95.3 [93.9, 96.8]	100.0 [100.0, 0.0]
Specificity in %		
Test 1	94.8 [93.3, 96.4]	71.0 [66.9, 75.1]
Test 2	96.8 [95.6, 98.0]	80.2 [76.6, 83.8]
Test 3	98.2 [97.2, 99.1]	87.9 [85.0, 90.9]
Test 4	98.8 [98.1, 99.6]	89.8 [87.1, 92.6]
Test 5	87.6 [85.3, 89.9]	59.4 [55.0, 63.9]
Test 6	77.0 [74.0, 80.0]	41.5 [37.0, 46.0]

In contrast, the other three starting value sets led to a significant deviation from the calculated parameter values of starting value set RI. These results were very similar to one another (Table 4). In total, 83.4–88.0% of animals were antibody positive, which is approximately 10% greater than that in the complete dataset, whereas the calculated values of the sensitivities and specificities were similar to those of the entire dataset. However, noticeable deviations of up to 11.6% were noted between the estimated parameter values of the different starting values.

**Table 4:** Resulting values for the prevalence and the test accuracies for the four starting value sets for the part of the sample that is vaccinated against genotype 1 of PRRSV with confidence limits reported in brackets (rounded to one digit).

Starting value sets	Set MI	Set MR	Set RI	Set RR
Prevalence in %	83.4 [80.0, 86.8]	88.0 [85.0, 90.9]	68.7 [64.6, 72.9]	87.4 [84.4, 90.4]
Sensitivity in %				
Test 1	91.8 [89.3, 94.3]	87.9 [85.0, 90.9]	100.0 [99.7, 100.0]	88.4 [85.5, 91.3]
Test 2	87.6 [84.6, 90.6]	83.4 [80.0, 86.7]	98.2 [97.0, 99.4]	83.9 [80.6, 87.2]
Test 3	84.2 [80.9, 87.5]	80.1 [76.5, 83.7]	97.4 [95.9, 98.8]	80.6 [77.1, 84.2]
Test 4	82.2 [78.8, 85.7]	78.1 [74.3, 81.8]	95.5 [93.6, 97.3]	78.6 [74.9, 82.3]
Test 5	94.6 [92.5, 96.6]	91.3 [88.8, 93.9]	100.0 [100.0, 0.0]	91.8 [89.3, 94.3]
Test 6	99.0 [98.1, 99.9]	96.9 [95.3, 98.4]	100.0 [100.0, 0.0]	96.1 [94.3, 97.9]
Specificity in %				
Test 1	92.5 [90.1, 94.9]	96.3 [94.6, 98.0]	71.0 [66.9, 75.1]	95.5 [93.7, 97.4]
Test 2	96.1 [94.3, 97.8]	96.9 [95.3, 98.4]	80.2 [76.6, 83.8]	97.0 [95.4, 98.5]
Test 3	96.8 [95.2, 98.4]	98.0 [96.7, 99.2]	87.9 [85.0, 90.9]	98.0 [96.7, 99.3]
Test 4	98.8 [97.7, 99.8]	99.0 [98.1, 99.9]	89.8 [87.1, 92.6]	98.9 [97.9, 99.8]
Test 5	83.3 [79.9, 86.7]	89.3 [86.5, 92.1]	59.4 [55.0, 63.9]	88.7 [85.8, 91.5]
Test 6	72.1 [68.1, 76.2]	83.7 [80.3, 87.0]	41.5 [37.0, 46.0]	74.6 [70.7, 78.5]

The dependencies of the tests were similar to those of the entire dataset. Only starting value set RI led to differing values. Thus, some dependency values were negative, which implies that one test has an increased probability of a positive result while that probability is reduced for the other test.

## Discussion

In this publication, we applied an iterative, frequentist latent class approach to real-life data of six conditionally dependent ELISA tests for PRRSV on pig farms with different vaccination statuses.

### Overall results

The analysis produced clear results for the entire dataset. All runs of the algorithm led to results with a maximum deviation of about three percentage points from the other results for all parameters under study, although the selected starting values differed strongly. Therefore, there seems to be only one maximum of the likelihood function, and these results can be considered reliable.

When interpreting the results, the methods of data collection must be considered. The samples were obtained during a voluntary examination that focused on the immune status after vaccination. As a result, only farms where the farmers had a high level of interest in the immune status of their animals and very few unvaccinated farms were included in the study. Therefore, this dataset is a convenience sample, and the proportion of unvaccinated farms is less than the proportion of unvaccinated farms in the total population, which is approximately 52% according to information from the Bavarian Animal Health Service. Thus, the calculated values for the test accuracies and the prevalence might not reflect the total population, as these values depend on the immune status of the animals examined [35]. However, this study provided a range of values of all the test accuracies indicating the relationship between the tests, which can serve as a basis for other investigations on the diagnostic tests used.

### Estimation difficulties in the vaccinated subgroup

The results for the analysis of the subset of the farms vaccinated against the EU strains of the virus were less clear, as the resulting values differed depending on the selected starting value. In particular, the prevalence and PrioCHECK values varied up to 42%. This finding suggests that the information on if and to which extent a vaccination was applied in the population under study is crucial for the evaluation. All four runs of the algorithm provided results that have the same value of the likelihood function. Therefore, each calculated parameter combination represents the unknown true parameters with the same probability under the given dataset. Thus, there is no unique solution, and the composition of the study population and the biological principles of the tests used must also be considered for interpretation.

Although the results of starting value sets MI, MR and RR had a high level of agreement, the calculated parameters for starting value set RI deviated from these values. In comparison, starting value set RI led to a lower prevalence, partially negative dependencies and 100% sensitivities for three of the tests. Perfect tests (i.e., perfect sensitivities) are not to be expected for the applied ELISA tests. Although a changed sensitivity of the tests used due to the selection of an equally vaccinated subgroup with less variation in genotype was possible, technical errors and an insufficient antibody concentration in the sample were still possible [36]. A reduced prevalence in a subgroup with more vaccinated animals is also very unlikely. Moreover, negative conditional dependencies of the tests imply that the probability of deviating results between two tests is increased compared to independent tests. Due to the same biological principle of the tests, this result is not plausible. Thus, the results of starting value sets MI, MR and RR seem to be more likely to represent the true parameter values. In that case, the proportion of animals with antibodies was 8–13% greater than that in the entire dataset. Therefore, vaccination against the European strain of the virus seems to have been successful, with a proportion of approximately 83–88% antibody-positive animals.

The reason that these problems exclusively occur in the vaccinated subgroup despite the same starting values may be the fact that the animals were all vaccinated against the same genotype of the virus. This feature causes a stronger homogeneity in the immune status of the animals and results in a homogenous dataset regarding positive test results in different diagnostic tests. This subsequently means that the statistical method has fewer deviating response patterns available for estimation, which increases the variation of the results from different starting values. As this was not predictable after the initial evaluation studies [25], it is not possible to indicate a percentage threshold of vaccinated individuals above which such problems can occur based on this study. This underlines the fact that prevaccination information is crucial for the evaluation.

The high proportion of vaccinated animals in this sample complicated the estimation for the statistical method used, but incorrect results can be discovered using several carefully selected starting values. If the results of all starting values agree (as with the entire dataset), then there is a clear indication that these are the true parameter values. If there are deviating results (as in the vaccinated subgroup), implausible results can be determined by considering the test principles used and the population examined. Hence, the method is also applicable in the case of a vaccinated population.

### Effect of starting values

In the vaccinated subgroup differences in the results may appear, due to poorly chosen starting values. This was previously shown in simulation studies [25]. Therefore, we used starting values that we considered similar to the true parameter values based on various sources. However, these values differ only slightly and were in part selected based on subjective evaluations. Since there was a possibility that these assumptions were wrong, we systematically varied the individual parameter groups of the researchers’ assessment (starting values RR) for the entire data set in a sensitivity analysis to check what effect changed starting values may have on the result (S6 Table). These analyses showed that deviating starting values lead to different results in some cases (S7 Table). For example, some changed starting values resulted in a prevalence of 0% or negative dependencies, which does not seem plausible under given circumstances. This underlines the conclusion made in the previous publication that the choice of starting values is important.

The assumption of independent tests seems to deviate greatly from the true parameter values, depending on the chosen initial values of test accuracies. Therefore, independent tests do not seem to be a good choice for starting values for this dataset. The result of starting value set RI, which assumed independent tests, deviates strongly from the other biologically more plausible results. Independence of the tests is an obvious and easy-to-implement choice for the starting values. However, in the case of several ELISA tests with the same biological principle, it would be a rather inapt assumption. Therefore, analyzing the biological test principles used combined with experience from application seems to be the more appropriate approach for identifying starting values for the dependency, as that led to values that were more similar to the outcomes. However, starting value set MI, which also assumed independence, did not lead to implausible results. This result indicates that the selected initial test accuracies also influence the result. Some starting parameter constellations of test accuracies were closer to the results and therefore better suited as a starting point for the algorithm. Experience from application on the test accuracies led to starting values that were similar to the outcome. Previous publications also provided information on the values of the test accuracies. However, clear differences exist between the animals examined in the different studies in terms of species, sex and immune history. The statistical methods used also differed between the various publications. These factors influence the calculated values for the test accuracies [8, 35], which led to different results in the examined studies. These differences made determining the starting values based on the results of previous publications challenging. However, considering these influences, we were able to determine well-fitted starting values. The values from the manufacturers’ studies were always greater than the values calculated in this study, which was probably also due to the different study situations.

Overall, the sources used for the starting values were useful for the application to our statistical method. Some values were closer to the results than others. Given that this difference is due to the composition of the population examined in this study, this may be different from other studies on other diseases.

### Accuracy and applicability of the tests examined

Overall, the presented method calculated predominantly high accuracies for the diagnostic tests used in the complete dataset. We determined values of 74.4–95.3% for the sensitivity and 76.9–99.1% for the specificity of the tests. However, none of these tests had the best values for sensitivity and specificity simultaneously. Thus, the method again shows that there is no general gold standard that is applicable for all situations. The optimal test depends on the objective of the investigation and whether the false-negative or false-positive results are to be minimized. If the user wants to have a high level of certainty with the positive results and thus reduce the possibility of false-positive results, then a test with high specificity, such as the Ingezim PRRS 2.0, may represent the optimal test. Conversely, if all positive animals are actually to be identified as such, a test with maximum sensitivity (e.g., PrioCheck) might be the right choice. The accuracy of the diagnosis can also be further increased if a sequential test procedure is used [8]. For example, if false-positive animals are to be excluded with maximum certainty, it may be helpful to first sample the animals with a test with an overall very high accuracy (e.g., PRRS X3). All individuals with a positive test are then also sampled with a test with maximum specificity (e.g., ID Screen PRRS indirect or Ingezim PRRS 2.0), and only observations with two positive test results are then considered positive. This process minimizes the risk of a false-positive assessment and therefore an unnecessary intervention. However, the dependency of the tests must be taken into account when combining them. If two tests have a very high dependency, the same factors lead to incorrect results. A high dependency increases the probability of matching false results [37].

## Conclusion

In this publication, we calculated the test accuracies of six ELISA tests used for the detection of PRRSV in pigs in southern Germany. Using the examined dataset as an example, we were able to demonstrate that the applied latent class approach is able to determine the parameter values of conditionally dependent tests with suitable starting values. Different methods of choosing starting values were shown.

None of the tests used had the best sensitivity and specificity simultaneously. Hence, the detection method must be chosen depending on the general fit-for-purpose concept and the sample population under study.

Here, the estimation of the parameters in a vaccinated subgroup was less precise, which suggests the need to take the heterogeneity and homogeneity of the immune status into account.

## Supporting information

S1 Table Starting values for the stepwise latent class algorithm for the complete data set. (DOCX)

S2 Table Starting values for the stepwise latent class algorithm for the vaccinated subgroup. (DOCX)

S3 Table Resulting values for the stepwise latent class algorithm for the complete data set for the five starting value sets. (DOCX)

S4 Table Resulting values for the stepwise latent class algorithm for the vaccinated subgroup for the three starting value sets. (DOCX)

S5 Table Studies evaluating the diagnostic test accuracies of the tests analyzed in this publication that were used as sources of information for suitable starting values for the iterative, frequentist latent class analysis. (DOCX)

S6 Table Starting values for the sensitivity analysis of the stepwise latent class algorithm. (DOCX)

S7 Table Resulting values of the stepwise latent class algorithm for the for the sensitivity analysis. (DOCX)

S8 Table Raw data of the study. (XLSX)

S1 File The R code of the algorithm. (R)
