# Supplementary material for: An intercomparison study of ELISAs for the detection of porcine reproductive and respiratory syndrome virus – evaluating six conditionally dependent tests
Source: PLoS One. 2022 Jan 25;17(1):e0262944. doi: 10.1371/journal.pone.0262944 (PMC8789123; doi:10.1371/journal.pone.0262944)
Supplement: S2 Table — (DOCX) [file pone.0262944.s002.docx]

**S2 Table. Starting values for the stepwise latent class algorithm for the vaccinated subgroup**

| **Parameter** | **Starting values MI** | **Starting values MR** | **Starting values RI** | **Starting values RR** |
| --- | --- | --- | --- | --- |
| Prevalence | 0.87 | 0.87 | 0.84 | 0.84 |
| Sensitivity 1 | 0.988 | 0.988 | 0.92 | 0.92 |
| Sensitivity 2 | 0.999 | 0.999 | 0.90 | 0.90 |
| Sensitivity 3 | 0.991 | 0.991 | 0.90 | 0.90 |
| Sensitivity 4 | 0.999 | 0.999 | 0.90 | 0.90 |
| Sensitivity 5 | 0.999 | 0.999 | 0.90 | 0.90 |
| Sensitvity 6 | 0.999 | 0.999 | 0.99 | 0.99 |
| Specifity 1 | 0.999 | 0.999 | 0.97 | 0.97 |
| Specifity 2 | 0.998 | 0.998 | 0.97 | 0.97 |
| Specifity 3 | 0.999 | 0.999 | 0.97 | 0.97 |
| Specifity 4 | 0.999 | 0.999 | 0.97 | 0.97 |
| Specifity 5 | 0.972 | 0.972 | 0.97 | 0.97 |
| Specifity 6 | 0.999 | 0.999 | 0.70 | 0.70 |
| ${}_{12}^{+}$^1^ | 0.000 | 0.016 | 0.000 | 0.016 |
| ${}_{13}^{+}$^1^ | 0.000 | 0.012 | 0.000 | 0.012 |
| ${}_{14}^{+}$^1^ | 0.000 | 0.008 | 0.000 | 0.008 |
| ${}_{15}^{+}$^1^ | 0.000 | 0.021 | 0.000 | 0.021 |
| ${}_{16}^{+}$^1^ | 0.000 | 0.030 | 0.000 | 0.030 |
| ${}_{23}^{+}$^1^ | 0.000 | 0.009 | 0.000 | 0.009 |
| ${}_{24}^{+}$^1^ | 0.000 | 0.008 | 0.000 | 0.008 |
| ${}_{25}^{+}$^1^ | 0.000 | 0.010 | 0.000 | 0.010 |
| ${}_{26}^{+}$^1^ | 0.000 | 0.013 | 0.000 | 0.013 |
| ${}_{34}^{+}$^1^ | 0.000 | 0.007 | 0.000 | 0.007 |
| ${}_{35}^{+}$^1^ | 0.000 | 0.008 | 0.000 | 0.008 |
| ${}_{36}^{+}$^1^ | 0.000 | 0.011 | 0.000 | 0.011 |
| ${}_{45}^{+}$^1^ | 0.000 | 0.001 | 0.000 | 0.001 |
| ${}_{46}^{+}$^1^ | 0.000 | 0.008 | 0.000 | 0.008 |
| ${}_{56}^{+}$^1^ | 0.000 | 0.032 | 0.000 | 0.032 |
| ${}_{123}^{+}$^1^ | 0.000 | 0.008 | 0.000 | 0.008 |
| ${}_{124}^{+}$^1^ | 0.000 | 0.007 | 0.000 | 0.007 |
| ${}_{125}^{+}$^1^ | 0.000 | 0.011 | 0.000 | 0.011 |
| ${}_{126}^{+}$^1^ | 0.000 | 0.009 | 0.000 | 0.009 |
| ${}_{134}^{+}$^1^ | 0.000 | 0.006 | 0.000 | 0.006 |
| ${}_{135}^{+}$^1^ | 0.000 | 0.007 | 0.000 | 0.007 |
| ${}_{136}^{+}$^1^ | 0.000 | 0.007 | 0.000 | 0.007 |
| ${}_{145}^{+}$^1^ | 0.000 | 0.006 | 0.000 | 0.006 |
| ${}_{146}^{+}$^1^ | 0.000 | 0.005 | 0.000 | 0.005 |
| ${}_{156}^{+}$^1^ | 0.000 | 0.010 | 0.000 | 0.010 |
| ${}_{234}^{+}$^1^ | 0.000 | 0.006 | 0.000 | 0.006 |
| ${}_{235}^{+}$^1^ | 0.000 | 0.007 | 0.000 | 0.007 |
| ${}_{236}^{+}$^1^ | 0.000 | 0.006 | 0.000 | 0.006 |
| ${}_{245}^{+}$^1^ | 0.000 | 0.007 | 0.000 | 0.007 |
| ${}_{246}^{+}$^1^ | 0.000 | 0.005 | 0.000 | 0.005 |
| ${}_{256}^{+}$^1^ | 0.000 | 0.007 | 0.000 | 0.007 |
| ${}_{345}^{+}$^1^ | 0.000 | 0.006 | 0.000 | 0.006 |
| ${}_{346}^{+}$^1^ | 0.000 | 0.005 | 0.000 | 0.005 |
| ${}_{356}^{+}$^1^ | 0.000 | 0.006 | 0.000 | 0.006 |
| ${}_{456}^{+}$^1^ | 0.000 | 0.007 | 0.000 | 0.007 |
| ${}_{1234}^{+}$^1^ | 0.000 | 0.006 | 0.000 | 0.006 |
| ${}_{1235}^{+}$^1^ | 0.000 | 0.006 | 0.000 | 0.006 |
| ${}_{1236}^{+}$^1^ | 0.000 | 0.006 | 0.000 | 0.006 |
| ${}_{1245}^{+}$^1^ | 0.000 | 0.006 | 0.000 | 0.006 |
| ${}_{1246}^{+}$^1^ | 0.000 | 0.005 | 0.000 | 0.005 |
| ${}_{1256}^{+}$^1^ | 0.000 | 0.006 | 0.000 | 0.006 |
| ${}_{1345}^{+}$^1^ | 0.000 | 0.006 | 0.000 | 0.006 |
| ${}_{1346}^{+}$^1^ | 0.000 | 0.005 | 0.000 | 0.005 |
| ${}_{1356}^{+}$^1^ | 0.000 | 0.005 | 0.000 | 0.005 |
| ${}_{1456}^{+}$^1^ | 0.000 | 0.005 | 0.000 | 0.005 |
| ${}_{2345}^{+}$^1^ | 0.000 | 0.005 | 0.000 | 0.005 |
| ${}_{2346}^{+}$^1^ | 0.000 | 0.005 | 0.000 | 0.005 |
| ${}_{2356}^{+}$^1^ | 0.000 | 0.005 | 0.000 | 0.005 |
| ${}_{2456}^{+}$^1^ | 0.000 | 0.005 | 0.000 | 0.005 |
| ${}_{3456}^{+}$^1^ | 0.000 | 0.005 | 0.000 | 0.005 |
| ${}_{12345}^{+}$^1^ | 0.000 | 0.005 | 0.000 | 0.005 |
| ${}_{12346}^{+}$^1^ | 0.000 | 0.004 | 0.000 | 0.004 |
| ${}_{12356}^{+}$^1^ | 0.000 | 0.005 | 0.000 | 0.005 |
| ${}_{12456}^{+}$^1^ | 0.000 | 0.004 | 0.000 | 0.004 |
| ${}_{13456}^{+}$^1^ | 0.000 | 0.004 | 0.000 | 0.004 |
| ${}_{23456}^{+}$^1^ | 0.000 | 0.004 | 0.000 | 0.004 |
| ${}_{123456}^{+}$^1^ | 0.000 | 0.004 | 0.000 | 0.004 |
| ${}_{12}^{-}$^1^ | 0.000 | 0.081 | 0.000 | 0.081 |
| ${}_{13}^{-}$^1^ | 0.000 | 0.085 | 0.000 | 0.085 |
| ${}_{14}^{-}$^1^ | 0.000 | 0.072 | 0.000 | 0.072 |
| ${}_{15}^{-}$^1^ | 0.000 | 0.050 | 0.000 | 0.050 |
| ${}_{16}^{-}$^1^ | 0.000 | 0.029 | 0.000 | 0.029 |
| ${}_{23}^{-}$^1^ | 0.000 | 0.102 | 0.000 | 0.102 |
| ${}_{24}^{-}$^1^ | 0.000 | 0.099 | 0.000 | 0.099 |
| ${}_{25}^{-}$^1^ | 0.000 | 0.058 | 0.000 | 0.058 |
| ${}_{26}^{-}$^1^ | 0.000 | 0.028 | 0.000 | 0.028 |
| ${}_{34}^{-}$^1^ | 0.000 | 0.122 | 0.000 | 0.122 |
| ${}_{35}^{-}$^1^ | 0.000 | 0.056 | 0.000 | 0.056 |
| ${}_{36}^{-}$^1^ | 0.000 | 0.034 | 0.000 | 0.034 |
| ${}_{45}^{-}$^1^ | 0.000 | 0.067 | 0.000 | 0.067 |
| ${}_{46}^{-}$^1^ | 0.000 | 0.030 | 0.000 | 0.030 |
| ${}_{56}^{-}$^1^ | 0.000 | 0.021 | 0.000 | 0.021 |
| ${}_{123}^{-}$^1^ | 0.000 | 0.049 | 0.000 | 0.049 |
| ${}_{124}^{-}$^1^ | 0.000 | 0.045 | 0.000 | 0.045 |
| ${}_{125}^{-}$^1^ | 0.000 | 0.033 | 0.000 | 0.033 |
| ${}_{126}^{-}$^1^ | 0.000 | 0.020 | 0.000 | 0.020 |
| ${}_{134}^{-}$^1^ | 0.000 | 0.041 | 0.000 | 0.041 |
| ${}_{135}^{-}$^1^ | 0.000 | 0.031 | 0.000 | 0.031 |
| ${}_{136}^{-}$^1^ | 0.000 | 0.020 | 0.000 | 0.020 |
| ${}_{145}^{-}$^1^ | 0.000 | 0.031 | 0.000 | 0.031 |
| ${}_{146}^{-}$^1^ | 0.000 | 0.019 | 0.000 | 0.019 |
| ${}_{156}^{-}$^1^ | 0.000 | 0.017 | 0.000 | 0.017 |
| ${}_{234}^{-}$^1^ | 0.000 | 0.053 | 0.000 | 0.053 |
| ${}_{235}^{-}$^1^ | 0.000 | 0.033 | 0.000 | 0.033 |
| ${}_{236}^{-}$^1^ | 0.000 | 0.017 | 0.000 | 0.017 |
| ${}_{245}^{-}$^1^ | 0.000 | 0.035 | 0.000 | 0.035 |
| ${}_{246}^{-}$^1^ | 0.000 | 0.017 | 0.000 | 0.017 |
| ${}_{256}^{-}$^1^ | 0.000 | 0.015 | 0.000 | 0.015 |
| ${}_{345}^{-}$^1^ | 0.000 | 0.032 | 0.000 | 0.032 |
| ${}_{346}^{-}$^1^ | 0.000 | 0.018 | 0.000 | 0.018 |
| ${}_{356}^{-}$^1^ | 0.000 | 0.013 | 0.000 | 0.013 |
| ${}_{456}^{-}$^1^ | 0.000 | 0.013 | 0.000 | 0.013 |
| ${}_{1234}^{-}$^1^ | 0.000 | 0.037 | 0.000 | 0.037 |
| ${}_{1235}^{-}$^1^ | 0.000 | 0.026 | 0.000 | 0.026 |
| ${}_{1236}^{-}$^1^ | 0.000 | 0.017 | 0.000 | 0.017 |
| ${}_{1245}^{-}$^1^ | 0.000 | 0.026 | 0.000 | 0.026 |
| ${}_{1246}^{-}$^1^ | 0.000 | 0.016 | 0.000 | 0.016 |
| ${}_{1256}^{-}$^1^ | 0.000 | 0.014 | 0.000 | 0.014 |
| ${}_{1345}^{-}$^1^ | 0.000 | 0.026 | 0.000 | 0.026 |
| ${}_{1346}^{-}$^1^ | 0.000 | 0.016 | 0.000 | 0.016 |
| ${}_{1356}^{-}$^1^ | 0.000 | 0.014 | 0.000 | 0.014 |
| ${}_{1456}^{-}$^1^ | 0.000 | 0.013 | 0.000 | 0.013 |
| ${}_{2345}^{-}$^1^ | 0.000 | 0.027 | 0.000 | 0.027 |
| ${}_{2346}^{-}$^1^ | 0.000 | 0.014 | 0.000 | 0.014 |
| ${}_{2356}^{-}$^1^ | 0.000 | 0.012 | 0.000 | 0.012 |
| ${}_{2456}^{-}$^1^ | 0.000 | 0.012 | 0.000 | 0.012 |
| ${}_{3456}^{-}$^1^ | 0.000 | 0.011 | 0.000 | 0.011 |
| ${}_{12345}^{-}$^1^ | 0.000 | 0.020 | 0.000 | 0.020 |
| ${}_{12346}^{-}$^1^ | 0.000 | 0.013 | 0.000 | 0.013 |
| ${}_{12356}^{-}$^1^ | 0.000 | 0.011 | 0.000 | 0.011 |
| ${}_{12456}^{-}$^1^ | 0.000 | 0.010 | 0.000 | 0.010 |
| ${}_{13456}^{-}$^1^ | 0.000 | 0.010 | 0.000 | 0.010 |
| ${}_{23456}^{-}$^1^ | 0.000 | 0.009 | 0.000 | 0.009 |
| ${}_{123456}^{-}$^1^ | 0.000 | 0.008 | 0.000 | 0.008 |

^1^ ${}_{ij}^{+}$ is the dependency of the sensitivities of test i and test j; ${}_{ij}^{-}$ is the dependency of the specifities of test i and test j
