# Supplementary material for: An intercomparison study of ELISAs for the detection of porcine reproductive and respiratory syndrome virus – evaluating six conditionally dependent tests
Source: PLoS One. 2022 Jan 25;17(1):e0262944. doi: 10.1371/journal.pone.0262944 (PMC8789123; doi:10.1371/journal.pone.0262944)
Supplement: S3 Table — (DOCX) [file pone.0262944.s003.docx]

**S3 Table. Resulting values for the stepwise latent class algorithm for the complete data set for the six starting value sets**

| **Parameter** | **Starting values MI** | **Starting values MR** | **Starting values PI** | **Starting values PR** | **Starting values RI** | **Starting values RR** |
| --- | --- | --- | --- | --- | --- | --- |
| Prevalence  lCl  uCl | 0.7545134  0.7247642 0.7842626 | 0.7666076  0.7373689 0.7958463 | 0.7567326  0.7270746 0.7863905 | 0.7679239  0.7387427 0.7971050 | 0.7529343  0.7231208 0.7827478 | 0.7637762  0.7344151 0.7931373 |
| Sensitivity 1  lCl  uCl | 0.8885030  0.8667465 0.9102595 | 0.8767316  0.8540074 0.8994557 | 0.8864680  0.8645391 0.9083970 | 0.8756724  0.8528646 0.8984801 | 0.8899512  0.8683189 0.9115836 | 0.8792851  0.8567648 0.9018053 |
| Sensitivity 2  lCl  uCl | 0.8436503  0.8185454 0.8687551 | 0.8309218  0.8050127 0.8568309 | 0.8415248  0.8162818 0.8667679 | 0.8297584 0.8037785 0.8557382 | 0.8451777  0.8201732 0.8701822 | 0.8335559  0.8078087 0.8593031 |
| Sensitivity 3  lCl  uCl | 0.7969744  0.7691692 0.8247796 | 0.7845615  0.7561428 0.8129801 | 0.7948701  0.7669581 0.8227820 | 0.7833689  0.7548934 0.8118444 | 0.7984812  0.7707532 0.8262092 | 0.7871817  0.7588893 0.8154741 |
| Sensitivity 4  lCl  uCl | 0.7561575  0.7264758 0.7858392 | 0.7450837  0.7149585 0.7752088 | 0.7540996  0.7243335 0.7838657 | 0.7438616  0.7136891 0.7740341 | 0.7576277  0.7280069 0.7872486 | 0.7476597  0.7176354 0.7776841 |
| Sensitivity 5  lCl  uCl | 0.9129749  0.8934908 0.9324589 | 0.9048626  0.8845814 0.9251439 | 0.9112788  0.8916240 0.9309335 | 0.9036336  0.8832356 0.9240316 | 0.9141841  0.8948231 0.9335452 | 0.9070473  0.8869761 0.9271186 |
| Sensitvity 6  lCl  uCl | 0.9525908  0.9379011 0.9672805 | 0.9498199  0.9347291 0.9649108 | 0.9513969  0.9365327 0.9662611 | 0.9490386  0.9338370 0.9642403 | 0.9534354  0.9388707 0.9680001 | 0.9513975  0.9365334 0.9662616 |
| Specifity 1  lCl  uCl | 0.9492908  0.9341248 0.9644568 | 0.9540399  0.9395654 0.9685143 | 0.9506034  0.9356246 0.9655822 | 0.9552470  0.9409549 0.9695392 | 0.9483496  0.9330511 0.9636481 | 0.9523384  0.9376116 0.9670652 |
| Specifity 2  lCl  uCl | 0.9684986  0.9564249 0.9805724 | 0.9687751  0.9567527 0.9807974 | 0.9692956  0.9573707 0.9812206 | 0.9694612  0.9575674 0.9813550 | 0.9679626  0.9557899 0.9801353 | 0.9677067  0.9554871 0.9799263 |
| Specifity 3  lCl  uCl | 0.9821027  0.9729384 0.9912670 | 0.9817019  0.9724375 0.9909664 | 0.9826638  0.9736417 0.9916859 | 0.9821019  0.9729374 0.9912664 | 0.9817152  0.9724540 0.9909764 | 0.9809894  0.9715496 0.9904291 |
| Specifity 4  lCl  uCl | 0.9883819  0.9809746 0.9957891 | 0.9905897  0.9839158 0.9972635 | 0.9887722  0.9814889 0.9960554 | 0.9907187  0.9840903 0.9973471 | 0.9881036  0.9806091 0.9955980 | 0.9901010  0.9832577 0.9969443 |
| Specifity 5  lCl  uCl | 0.8775750  0.8549178 0.9002321 | 0.8918949  0.8704310 0.9133587 | 0.8795105  0.8570084 0.9020126 | 0.8923472  0.8709229 0.9137716 | 0.8762073  0.8534418 0.8989729 | 0.8894086  0.8677296 0.9110876 |
| Specifity 6  lCl  uCl | 0.7713395  0.7423096 0.8003695 | 0.7997516  0.7720892 0.8274140 | 0.7742294  0.7453295 0.8031293 | 0.8014178  0.7738420 0.8289935 | 0.7692863  0.7401652 0.7984075 | 0.7958679  0.7680064 0.8237294 |
| ${}_{12}^{+}$^1^ | 0.0160583 | 0.0157224 | 0.0155491 | 0.0152712 | 0.0164139 | 0.0164217 |
| ${}_{13}^{+}$^1^ | 0.0127707 | 0.0130732 | 0.0123663 | 0.0127620 | 0.0130512 | 0.0136039 |
| ${}_{14}^{+}$^1^ | 0.0078364 | 0.0071215 | 0.0075776 | 0.0069914 | 0.0080154 | 0.0074637 |
| ${}_{15}^{+}$^1^ | 0.0215780 | 0.0195980 | 0.0210145 | 0.0191953 | 0.0219715 | 0.0203212 |
| ${}_{16}^{+}$^1^ | 0.0308721 | 0.0289343 | 0.0301886 | 0.0281790 | 0.0313640 | 0.0298910 |
| ${}_{23}^{+}$^1^ | 0.0089938 | 0.0090419 | 0.0086850 | 0.0088119 | 0.0092127 | 0.0094733 |
| ${}_{24}^{+}$^1^ | 0.0080675 | 0.0072983 | 0.0078000 | 0.0071637 | 0.0082591 | 0.0076593 |
| ${}_{25}^{+}$^1^ | 0.0141282 | 0.0136593 | 0.0137060 | 0.0133439 | 0.0144215 | 0.0142361 |
| ${}_{26}^{+}$^1^ | 0.0128971 | 0.0133050 | 0.0125348 | 0.0129251 | 0.0131537 | 0.0138397 |
| ${}_{34}^{+}$^1^ | 0.0071134 | 0.0066447 | 0.0068761 | 0.0065212 | 0.0072823 | 0.0069677 |
| ${}_{35}^{+}$^1^ | 0.0080491 | 0.0079772 | 0.0077849 | 0.0078062 | 0.0082302 | 0.0083386 |
| ${}_{36}^{+}$^1^ | 0.0116525 | 0.0122630 | 0.0113183 | 0.0120174 | 0.0118839 | 0.0127084 |
| ${}_{45}^{+}$^1^ | 0.0101958 | 0.0083930 | 0.0098750 | 0.0082822 | 0.0104238 | 0.0088043 |
| ${}_{46}^{+}$^1^ | 0.0083727 | 0.0070406 | 0.0081279 | 0.0069618 | 0.0085494 | 0.0073655 |
| ${}_{56}^{+}$^1^ | 0.0329482 | 0.0295674 | 0.0322496 | 0.0291915 | 0.0334719 | 0.0306336 |
| ${}_{123}^{+}$^1^ | 0.0083832 | 0.0084755 | 0.0081127 | 0.0082766 | 0.0085741 | 0.0088549 |
| ${}_{124}^{+}$^1^ | 0.0068689 | 0.0063724 | 0.0066559 | 0.0062621 | 0.0070204 | 0.0066644 |
| ${}_{125}^{+}$^1^ | 0.0108430 | 0.0105979 | 0.0105335 | 0.0103551 | 0.0110550 | 0.0110236 |
| ${}_{126}^{+}$^1^ | 0.0094003 | 0.0097685 | 0.0091566 | 0.0095190 | 0.0095705 | 0.0101277 |
| ${}_{134}^{+}$^1^ | 0.0064745 | 0.0061039 | 0.0062711 | 0.0059971 | 0.0066185 | 0.0063835 |
| ${}_{135}^{+}$^1^ | 0.0070052 | 0.0069975 | 0.0067916 | 0.0068550 | 0.0071505 | 0.0072941 |
| ${}_{136}^{+}$^1^ | 0.0072547 | 0.0076639 | 0.0070510 | 0.0075010 | 0.0073970 | 0.0079525 |
| ${}_{145}^{+}$^1^ | 0.0066263 | 0.0061672 | 0.0064286 | 0.0060606 | 0.0067618 | 0.0064371 |
| ${}_{146}^{+}$^1^ | 0.0051269 | 0.0049601 | 0.0049907 | 0.0048864 | 0.0052217 | 0.0051559 |
| ${}_{156}^{+}$^1^ | 0.0097529 | 0.0095597 | 0.0095638 | 0.0093995 | 0.0098804 | 0.0098247 |
| ${}_{234}^{+}$^1^ | 0.0063089 | 0.0059637 | 0.0061123 | 0.0058559 | 0.0064540 | 0.0062402 |
| ${}_{235}^{+}$^1^ | 0.0069300 | 0.0069143 | 0.0067142 | 0.0067643 | 0.0070818 | 0.0072185 |
| ${}_{236}^{+}$^1^ | 0.0066345 | 0.0069127 | 0.0064395 | 0.0067571 | 0.0067749 | 0.0071968 |
| ${}_{245}^{+}$^1^ | 0.0069156 | 0.0063808 | 0.0067063 | 0.0062687 | 0.0070651 | 0.0066714 |
| ${}_{246}^{+}$^1^ | 0.0055026 | 0.0052415 | 0.0053506 | 0.0051593 | 0.0056140 | 0.0054626 |
| ${}_{256}^{+}$^1^ | 0.0068492 | 0.0070009 | 0.0066869 | 0.0068607 | 0.0069614 | 0.0072409 |
| ${}_{345}^{+}$^1^ | 0.0061491 | 0.0058513 | 0.0059602 | 0.0057467 | 0.0062829 | 0.0061146 |
| ${}_{346}^{+}$^1^ | 0.0052223 | 0.0050585 | 0.0050743 | 0.0049785 | 0.0053301 | 0.0052715 |
| ${}_{356}^{+}$^1^ | 0.0057426 | 0.0059460 | 0.0055897 | 0.0058393 | 0.0058478 | 0.0061641 |
| ${}_{456}^{+}$^1^ | 0.0069649 | 0.0060012 | 0.0067865 | 0.0059414 | 0.0070928 | 0.0062477 |
| ${}_{1234}^{+}$^1^ | 0.0060074 | 0.0057037 | 0.0058277 | 0.0056075 | 0.0061400 | 0.0059583 |
| ${}_{1235}^{+}$^1^ | 0.0066324 | 0.0066490 | 0.0064333 | 0.0065123 | 0.0067721 | 0.0069311 |
| ${}_{1236}^{+}$^1^ | 0.0064515 | 0.0067580 | 0.0062670 | 0.0066114 | 0.0065837 | 0.0070275 |
| ${}_{1245}^{+}$^1^ | 0.0060657 | 0.0057158 | 0.0058892 | 0.0056183 | 0.0061909 | 0.0059628 |
| ${}_{1246}^{+}$^1^ | 0.0047916 | 0.0046742 | 0.0046649 | 0.0046026 | 0.0048841 | 0.0048605 |
| ${}_{1256}^{+}$^1^ | 0.0064590 | 0.0066298 | 0.0062985 | 0.0064930 | 0.0065698 | 0.0068653 |
| ${}_{1345}^{+}$^1^ | 0.0057306 | 0.0054879 | 0.0055614 | 0.0053936 | 0.0058502 | 0.0057251 |
| ${}_{1346}^{+}$^1^ | 0.0049015 | 0.0047741 | 0.0047674 | 0.0047014 | 0.0049990 | 0.0049678 |
| ${}_{1356}^{+}$^1^ | 0.0056442 | 0.0058104 | 0.0054900 | 0.0057019 | 0.0057510 | 0.0060306 |
| ${}_{1456}^{+}$^1^ | 0.0047007 | 0.0045768 | 0.0045801 | 0.0045065 | 0.0047847 | 0.0047516 |
| ${}_{2345}^{+}$^1^ | 0.0055477 | 0.0053298 | 0.0053862 | 0.0052357 | 0.0056668 | 0.0055620 |
| ${}_{2346}^{+}$^1^ | 0.0047447 | 0.0046366 | 0.0046171 | 0.0045638 | 0.0048422 | 0.0048265 |
| ${}_{2356}^{+}$^1^ | 0.0052913 | 0.0054652 | 0.0051466 | 0.0053573 | 0.0053952 | 0.0056788 |
| ${}_{2456}^{+}$^1^ | 0.0048809 | 0.0047206 | 0.0047546 | 0.0046473 | 0.0049733 | 0.0056788 |
| ${}_{3456}^{+}$^1^ | 0.0046350 | 0.0045586 | 0.0045120 | 0.0044879 | 0.0047246 | 0.0047394 |
| ${}_{12345}^{+}$^1^ | 0.0052804 | 0.0050957 | 0.0051334 | 0.0050119 | 0.0053887 | 0.0053087 |
| ${}_{12346}^{+}$^1^ | 0.0045151 | 0.0044321 | 0.0043995 | 0.0043680 | 0.0046036 | 0.0046058 |
| ${}_{12356}^{+}$^1^ | 0.0050298 | 0.0052213 | 0.0048992 | 0.0051249 | 0.0051234 | 0.0054154 |
| ${}_{12456}^{+}$^1^ | 0.0042258 | 0.0041885 | 0.0041225 | 0.0041257 | 0.0043011 | 0.0043442 |
| ${}_{13456}^{+}$^1^ | 0.0043098 | 0.0042676 | 0.0042010 | 0.0042045 | 0.0043889 | 0.0044288 |
| ${}_{23456}^{+}$^1^ | 0.0041700 | 0.0041420 | 0.0040666 | 0.0040788 | 0.0042492 | 0.0042999 |
| ${}_{123456}^{+}$^1^ | 0.0039697 | 0.0039606 | 0.0038762 | 0.0039050 | 0.0040414 | 0.0041047 |
| ${}_{12}^{-}$^1^ | 0.0804363 | 0.0888623 | 0.0818446 | 0.0895466 | 0.0794234 | 0.0871423 |
| ${}_{13}^{-}$^1^ | 0.0836392 | 0.0915566 | 0.0849911 | 0.0922259 | 0.0826717 | 0.0899027 |
| ${}_{14}^{-}$^1^ | 0.0705120 | 0.0778084 | 0.0718170 | 0.0784721 | 0.0695807 | 0.0762048 |
| ${}_{15}^{-}$^1^ | 0.0485143 | 0.0542293 | 0.0497000 | 0.0550206 | 0.0476705 | 0.0527314 |
| ${}_{16}^{-}$^1^ | 0.0283202 | 0.0301515 | 0.0292386 | 0.0307519 | 0.0276676 | 0.0289379 |
| ${}_{23}^{-}$^1^ | 0.1009448 | 0.1093372 | 0.1022793 | 0.1100281 | 0.0999770 | 0.1077262 |
| ${}_{24}^{-}$^1^ | 0.0978313 | 0.1054391 | 0.0991301 | 0.1061349 | 0.0968913 | 0.1038674 |
| ${}_{25}^{-}$^1^ | 0.0563866 | 0.0622800 | 0.0575692 | 0.0631081 | 0.0555339 | 0.0607915 |
| ${}_{26}^{-}$^1^ | 0.0272217 | 0.0290233 | 0.0280770 | 0.0295793 | 0.0266073 | 0.0278969 |
| ${}_{34}^{-}$^1^ | 0.1202998 | 0.1272379 | 0.1215088 | 0.1279145 | 0.1194288 | 0.1257664 |
| ${}_{35}^{-}$^1^ | 0.0553636 | 0.0609566 | 0.0564846 | 0.0617613 | 0.0545598 | 0.0595365 |
| ${}_{36}^{-}$^1^ | 0.0335389 | 0.0352238 | 0.0343786 | 0.0357656 | 0.0329413 | 0.0341226 |
| ${}_{45}^{-}$^1^ | 0.0658047 | 0.0708853 | 0.0669047 | 0.0716833 | 0.0650165 | 0.0694970 |
| ${}_{46}^{-}$^1^ | 0.0294466 | 0.0310463 | 0.0302588 | 0.0315735 | 0.0288687 | 0.0299829 |
| ${}_{56}^{-}$^1^ | 0.0204275 | 0.0205425 | 0.0211785 | 0.0211904 | 0.0198847 | 0.0194923 |
| ${}_{123}^{-}$^1^ | 0.0484458 | 0.0519566 | 0.0490241 | 0.0521798 | 0.0480207 | 0.0513233 |
| ${}_{124}^{-}$^1^ | 0.0446394 | 0.0478864 | 0.0452097 | 0.0481181 | 0.0442220 | 0.0472536 |
| ${}_{125}^{-}$^1^ | 0.0325730 | 0.0354490 | 0.0331894 | 0.0358442 | 0.0321240 | 0.0347171 |
| ${}_{126}^{-}$^1^ | 0.0194242 | 0.0200989 | 0.0199577 | 0.0204462 | 0.0190360 | 0.0194060 |
| ${}_{134}^{-}$^1^ | 0.0404228 | 0.0430980 | 0.0409152 | 0.0432997 | 0.0400649 | 0.0425563 |
| ${}_{135}^{-}$^1^ | 0.0307727 | 0.0333907 | 0.0313551 | 0.0337725 | 0.0303525 | 0.0326988 |
| ${}_{136}^{-}$^1^ | 0.0190785 | 0.0195724 | 0.0195931 | 0.0199068 | 0.0187091 | 0.0189092 |
| ${}_{145}^{-}$^1^ | 0.0305481 | 0.0330029 | 0.0311068 | 0.0333656 | 0.0301454 | 0.0323417 |
| ${}_{146}^{-}$^1^ | 0.0187855 | 0.0193427 | 0.0192841 | 0.0196637 | 0.0184272 | 0.0186991 |
| ${}_{156}^{-}$^1^ | 0.0160575 | 0.0156599 | 0.0165772 | 0.0161285 | 0.0156763 | 0.0149306 |
| ${}_{234}^{-}$^1^ | 0.0526338 | 0.0550674 | 0.0530182 | 0.0552208 | 0.0523454 | 0.0546738 |
| ${}_{235}^{-}$^1^ | 0.0322814 | 0.0348486 | 0.0327967 | 0.0352051 | 0.0319015 | 0.0342427 |
| ${}_{236}^{-}$^1^ | 0.0165794 | 0.0170389 | 0.0170110 | 0.0173115 | 0.0162641 | 0.0164843 |
| ${}_{245}^{-}$^1^ | 0.0341234 | 0.0363726 | 0.0346180 | 0.0367159 | 0.0337592 | 0.0357966 |
| ${}_{246}^{-}$^1^ | 0.0169468 | 0.0174061 | 0.0173680 | 0.0176725 | 0.0166391 | 0.0168669 |
| ${}_{256}^{-}$^1^ | 0.0146511 | 0.0141594 | 0.0151085 | 0.0145775 | 0.0143103 | 0.0135131 |
| ${}_{345}^{-}$^1^ | 0.0313945 | 0.0333127 | 0.0318146 | 0.0336152 | 0.0310871 | 0.0328227 |
| ${}_{346}^{-}$^1^ | 0.0178064 | 0.0180837 | 0.0181825 | 0.0183192 | 0.0175352 | 0.0176049 |
| ${}_{356}^{-}$^1^ | 0.0130313 | 0.0125064 | 0.0134565 | 0.0128926 | 0.0127185 | 0.0119065 |
| ${}_{456}^{-}$^1^ | 0.0128839 | 0.0123522 | 0.0132861 | 0.0127201 | 0.0125878 | 0.0117866 |
| ${}_{1234}^{-}$^1^ | 0.0364099 | 0.0390985 | 0.0368649 | 0.0392825 | 0.0360770 | 0.0385927 |
| ${}_{1235}^{-}$^1^ | 0.0259208 | 0.0281754 | 0.0263915 | 0.0284794 | 0.0255765 | 0.0276185 |
| ${}_{1236}^{-}$^1^ | 0.0162598 | 0.0166903 | 0.0166811 | 0.0169641 | 0.0159527 | 0.0161445 |
| ${}_{1245}^{-}$^1^ | 0.0260387 | 0.0281557 | 0.0264929 | 0.0284452 | 0.0257069 | 0.0276204 |
| ${}_{1246}^{-}$^1^ | 0.0153434 | 0.0157627 | 0.0157375 | 0.0160175 | 0.0150557 | 0.0152513 |
| ${}_{1256}^{-}$^1^ | 0.0130928 | 0.0126735 | 0.0134954 | 0.0130438 | 0.0127918 | 0.0121013 |
| ${}_{1345}^{-}$^1^ | 0.0252551 | 0.0272686 | 0.0257011 | 0.0275605 | 0.0249327 | 0.0267398 |
| ${}_{1346}^{-}$^1^ | 0.0157147 | 0.0160625 | 0.0161101 | 0.0163171 | 0.0154302 | 0.0155528 |
| ${}_{1356}^{-}$^1^ | 0.0132412 | 0.0128136 | 0.0136446 | 0.0131796 | 0.0129444 | 0.0122469 |
| ${}_{1456}^{-}$^1^ | 0.0123708 | 0.0119651 | 0.0127462 | 0.0123069 | 0.0120943 | 0.0114360 |
| ${}_{2345}^{-}$^1^ | 0.0264720 | 0.0284765 | 0.0268711 | 0.0287535 | 0.0261775 | 0.0280065 |
| ${}_{2346}^{-}$^1^ | 0.0139494 | 0.0143204 | 0.0142909 | 0.0145350 | 0.0136999 | 0.0138806 |
| ${}_{2356}^{-}$^1^ | 0.0114671 | 0.0110494 | 0.0118194 | 0.0113723 | 0.0112036 | 0.0105473 |
| ${}_{2456}^{-}$^1^ | 0.0113951 | 0.0109513 | 0.0117309 | 0.0112609 | 0.0111439 | 0.0104742 |
| ${}_{3456}^{-}$^1^ | 0.0105111 | 0.0100947 | 0.0108344 | 0.0103888 | 0.0102728 | 0.0096370 |
| ${}_{12345}^{-}$^1^ | 0.0196196 | 0.0209563 | 0.0199111 | 0.0211396 | 0.0194037 | 0.0206288 |
| ${}_{12346}^{-}$^1^ | 0.0122733 | 0.0123793 | 0.0125513 | 0.0125577 | 0.0120687 | 0.0120239 |
| ${}_{12356}^{-}$^1^ | 0.0105267 | 0.0100189 | 0.0108197 | 0.0102937 | 0.0103058 | 0.0096025 |
| ${}_{12456}^{-}$^1^ | 0.0098432 | 0.0093679 | 0.0101159 | 0.0096249 | 0.0096371 | 0.0089786 |
| ${}_{13456}^{-}$^1^ | 0.0099003 | 0.0094065 | 0.0101710 | 0.0096584 | 0.0096992 | 0.0090250 |
| ${}_{23456}^{-}$^1^ | 0.0085480 | 0.0080781 | 0.0087832 | 0.0082997 | 0.0083699 | 0.0077412 |
| ${}_{123456}^{-}$^1^ | 0.0079618 | 0.0074703 | 0.0081616 | 0.0076630 | 0.0078094 | 0.0071838 |
| Log-Likelihood | -1402.758 | -1402.758 | -1402.758 | -1402.758 | -1402.758 | -1402.758 |
| Iterations^2^ | 13 | 11 | 12 | 10 | 11 | 10 |

lCl: lower confidence limit, uCl: upper confidence limit

^1^ ${}_{ij}^{+}$ is the dependency of the sensitivities of test i and test j; ${}_{ij}^{-}$ is the dependency of the specifities of test i and test j

^2^ The number of the iterations the whole algorithm, not the ones of the EM algorithm performed at each step
