# Supplementary material for: An intercomparison study of ELISAs for the detection of porcine reproductive and respiratory syndrome virus – evaluating six conditionally dependent tests
Source: PLoS One. 2022 Jan 25;17(1):e0262944. doi: 10.1371/journal.pone.0262944 (PMC8789123; doi:10.1371/journal.pone.0262944)
Supplement: S4 Table — (DOCX) [file pone.0262944.s004.docx]

**S4 Table. Resulting values for the stepwise latent class algorithm for the vaccinated subgroup for the four starting value sets**

| **Parameter** | **Starting values MI** | **Starting values MR** | **Starting values RI** | **Starting values RR** |
| --- | --- | --- | --- | --- |
| Prevalence  lCl  uCl | 0.8340011  0.8002903 0.8677119 | 0.8797012  0.8502278 0.9091747 | 0.6874968  0.6455020 0.7294916 | 0.8738472  0.8437658 0.9039287 |
| Sensitivity 1  lCl  uCl | 0.9177018  0.8928029 0.9426006 | 0.8791367  0.8496036 0.9086698 | 0.9995038  0.9974862 1.0015215 | 0.8836254  0.8545721 0.9126788 |
| Sensitivity 2  lCl  uCl | 0.8761292  0.8462822 0.9059763 | 0.8337009  0.7999657 0.8674361 | 0.9823997  0.9704863 0.9943132 | 0.8392046  0.8059230 0.8724862 |
| Sensitivity 3  lCl  uCl | 0.8415881  0.8085072 0.8746690 | 0.8011879  0.7650284 0.8373473 | 0.9738739  0.9594221 0.9883257 | 0.8064909  0.7706991 0.8422827 |
| Sensitivity 4  lCl  uCl | 0.8224948  0.7878765 0.8571130 | 0.7807156  0.7432284 0.8182029 | 0.9545927  0.9357300 0.9734555 | 0.7857367  0.7485622 0.8229112 |
| Sensitivity 5  lCl  uCl | 0.9455100  0.9249452 0.9660748 | 0.9132076 0.8877006 0.9387145 | 1.0027371  -^3^  -^3^ | 0.9177181  0.8928215 0.9426147 |
| Sensitvity 6  lCl  uCl | 0.9898129  0.9807151 0.9989107 | 0.9687037  0.9529285 0.9844789 | 1.0021484  -^3^  -^3^ | 0.9609851  0.9434419 0.9785282 |
| Specifity 1  lCl  uCl | 0.9252195  0.9013881 0.9490509 | 0.9634234  0.9464158 0.9804310 | 0.7100122  0.6689014 0.7511230 | 0.9554180  0.9367193 0.9741166 |
| Specifity 2  lCl  uCl | 0.9609231  0.9433666 0.9784796 | 0.9686463  0.9528571 0.9844355 | 0.8022970  0.7662136 0.8383804 | 0.9695379  0.9539676 0.9851081 |
| Specifity 3  lCl  uCl | 0.9675930  0.9515495 0.9836365 | 0.9795592  0.9667389 0.9923795 | 0.8792660  0.8497465 0.9087854 | 0.9800632  0.9673987 0.9927277 |
| Specifity 4  lCl  uCl | 0.9875143  0.9774540 0.9975746 | 0.9897116  0.9805692 0.9988540 | 0.8983858  0.8710116 0.9257600 | 0.9887411  0.9791819 0.9983003 |
| Specifity 5  lCl  uCl | 0.8332342  0.7994611 0.8670072 | 0.8928543  0.8648316 0.9208771 | 0.5940496  0.5495577 0.6385415 | 0.8866938  0.8579762 0.9154113 |
| Specifity 6  lCl  uCl | 0.7211439  0.6805151 0.7617727 | 0.8368640  0.8033879 0.8703402 | 0.4149786  0.3703378 0.4596194 | 0.7460164  0.7065788 0.7854539 |
| ${}_{12}^{+}$^1^ | 0.0159569 | 0.0108192 | 0.0980023 | 0.0143303 |
| ${}_{13}^{+}$^1^ | 0.0228745 | 0.0132577 | 0.0738307 | 0.0154019 |
| ${}_{14}^{+}$^1^ | 0.0059857 | 0.0066310 | 0.0380955 | 0.0079797 |
| ${}_{15}^{+}$^1^ | 0.0220352 | 0.0140534 | 0.1130476 | 0.0190629 |
| ${}_{16}^{+}$^1^ | 0.0464414 | 0.0254506 | 0.1150169 | 0.0290739 |
| ${}_{23}^{+}$^1^ | 0.0072372 | 0.0066216 | 0.0429163 | 0.0078226 |
| ${}_{24}^{+}$^1^ | 0.0064075 | 0.0069675 | 0.0496910 | 0.0081000 |
| ${}_{25}^{+}$^1^ | 0.0130288 | 0.0091876 | 0.0905796 | 0.0127718 |
| ${}_{26}^{+}$^1^ | 0.0175709 | 0.0129860 | 0.0753081 | 0.0139250 |
| ${}_{34}^{+}$^1^ | 0.0058696 | 0.0082833 | 0.0497412 | 0.0078978 |
| ${}_{35}^{+}$^1^ | 0.0067213 | 0.0079095 | 0.0461127 | 0.0079197 |
| ${}_{36}^{+}$^1^ | 0.0158840 | 0.0119472 | 0.0447802 | 0.0106882 |
| ${}_{45}^{+}$^1^ | 0.0104035 | 0.0091860 | 0.0603639 | 0.0099832 |
| ${}_{46}^{+}$^1^ | 0.0090040 | 0.0086100 | 0.0421677 | 0.0083993 |
| ${}_{56}^{+}$^1^ | 0.0657057 | 0.0353451 | 0.1282003 | 0.0351269 |
| ${}_{123}^{+}$^1^ | 0.0064567 | 0.0063600 | 0.0209900 | 0.0072992 |
| ${}_{124}^{+}$^1^ | 0.0046992 | 0.0055926 | 0.0165465 | 0.0069074 |
| ${}_{125}^{+}$^1^ | 0.0102060 | 0.0084210 | 0.0303162 | 0.0106337 |
| ${}_{126}^{+}$^1^ | 0.0104858 | 0.0087410 | 0.0198826 | 0.0101974 |
| ${}_{134}^{+}$^1^ | 0.0048919 | 0.0055037 | 0.0268194 | 0.0071698 |
| ${}_{135}^{+}$^1^ | 0.0062566 | 0.0056152 | 0.0188130 | 0.0073419 |
| ${}_{136}^{+}$^1^ | 0.0080645 | 0.0056043 | 0.0129731 | 0.0069120 |
| ${}_{145}^{+}$^1^ | 0.0047123 | 0.0057759 | 0.0111434 | 0.0068609 |
| ${}_{146}^{+}$^1^ | 0.0037367 | 0.0052874 | 0.0041215 | 0.0056257 |
| ${}_{156}^{+}$^1^ | 0.0122255 | 0.0110208 | 0.0118962 | 0.0131294 |
| ${}_{234}^{+}$^1^ | 0.0045518 | 0.0054894 | 0.0258303 | 0.0068677 |
| ${}_{235}^{+}$^1^ | 0.0064006 | 0.0060486 | 0.0196214 | 0.0069789 |
| ${}_{236}^{+}$^1^ | 0.0049422 | 0.0054377 | 0.0097692 | 0.0056857 |
| ${}_{245}^{+}$^1^ | 0.0051763 | 0.0061265 | 0.0203148 | 0.0070384 |
| ${}_{246}^{+}$^1^ | 0.0044014 | 0.0056973 | 0.0129683 | 0.0058860 |
| ${}_{256}^{+}$^1^ | 0.0085973 | 0.0080006 | 0.0149769 | 0.0094550 |
| ${}_{345}^{+}$^1^ | 0.0048068 | 0.0073144 | 0.0248630 | 0.0069138 |
| ${}_{346}^{+}$^1^ | 0.0040345 | 0.0068091 | 0.0160912 | 0.0057715 |
| ${}_{356}^{+}$^1^ | 0.0039661 | 0.0064495 | 0.0058181 | 0.0056821 |
| ${}_{456}^{+}$^1^ | 0.0066821 | 0.0073238 | 0.0120228 | 0.0070521 |
| ${}_{1234}^{+}$^1^ | 0.0042647 | 0.0053849 | 0.0213724 | 0.0065847 |
| ${}_{1235}^{+}$^1^ | 0.0059525 | 0.0058900 | 0.0180911 | 0.0066943 |
| ${}_{1236}^{+}$^1^ | 0.0049600 | 0.0054847 | 0.0119670 | 0.0055770 |
| ${}_{1245}^{+}$^1^ | 0.0039541 | 0.0050061 | 0.0126647 | 0.0061482 |
| ${}_{1246}^{+}$^1^ | 0.0034016 | 0.0046835 | 0.0089786 | 0.0051586 |
| ${}_{1256}^{+}$^1^ | 0.0073709 | 0.0070185 | 0.0187106 | 0.0079217 |
| ${}_{1345}^{+}$^1^ | 0.0042360 | 0.0049781 | 0.0184771 | 0.0064285 |
| ${}_{1346}^{+}$^1^ | 0.0036330 | 0.0046624 | 0.0129245 | 0.0054003 |
| ${}_{1356}^{+}$^1^ | 0.0057857 | 0.0052469 | 0.0135686 | 0.0059520 |
| ${}_{1456}^{+}$^1^ | 0.0034440 | 0.0048413 | 0.0081824 | 0.0051307 |
| ${}_{2345}^{+}$^1^ | 0.0037881 | 0.0048998 | 0.0159412 | 0.0060891 |
| ${}_{2346}^{+}$^1^ | 0.0032860 | 0.0045950 | 0.0115360 | 0.0051251 |
| ${}_{2356}^{+}$^1^ | 0.0045928 | 0.0050381 | 0.0104453 | 0.0051934 |
| ${}_{2456}^{+}$^1^ | 0.0037428 | 0.0051238 | 0.0107279 | 0.0052516 |
| ${}_{3456}^{+}$^1^ | 0.0034774 | 0.0061229 | 0.0116709 | 0.0051603 |
| ${}_{12345}^{+}$^1^ | 0.0035464 | 0.0048058 | 0.0121250 | 0.0058361 |
| ${}_{12346}^{+}$^1^ | 0.0030717 | 0.0045048 | 0.0085382 | 0.0049108 |
| ${}_{12356}^{+}$^1^ | 0.0042471 | 0.0049130 | 0.0064641 | 0.0049805 |
| ${}_{12456}^{+}$^1^ | 0.0028514 | 0.0041898 | 0.0046327 | 0.0045868 |
| ${}_{13456}^{+}$^1^ | 0.0030388 | 0.0041603 | 0.0070821 | 0.0047904 |
| ${}_{23456}^{+}$^1^ | 0.0027321 | 0.0041007 | 0.0063813 | 0.0045427 |
| ${}_{123456}^{+}$^1^ | 0.0025580 | 0.0040219 | 0.0051376 | 0.0043540 |
| ${}_{12}^{-}$^1^ | 0.0581897 | 0.0864124 | -0.0020096 | 0.0826783 |
| ${}_{13}^{-}$^1^ | 0.0655521 | 0.0928564 | -0.0003273 | 0.0894910 |
| ${}_{14}^{-}$^1^ | 0.0534255 | 0.0802348 | -0.0026961 | 0.0771694 |
| ${}_{15}^{-}$^1^ | 0.0349634 | 0.0569859 | -0.0037895 | 0.0536542 |
| ${}_{16}^{-}$^1^ | 0.0082767 | 0.0257903 | -0.0028363 | 0.0326335 |
| ${}_{23}^{-}$^1^ | 0.0731372 | 0.1010337 | -0.0018495 | 0.0971081 |
| ${}_{24}^{-}$^1^ | 0.0773753 | 0.1059528 | 0.0001910 | 0.1022971 |
| ${}_{25}^{-}$^1^ | 0.0413796 | 0.0652149 | -0.0029091 | 0.0613260 |
| ${}_{26}^{-}$^1^ | 0.0084748 | 0.0254751 | -0.0021574 | 0.0315668 |
| ${}_{34}^{-}$^1^ | 0.1059088 | 0.1311717 | 0.0118620 | 0.1280478 |
| ${}_{35}^{-}$^1^ | 0.0396468 | 0.0612354 | -0.0034569 | 0.0579875 |
| ${}_{36}^{-}$^1^ | 0.0075013 | 0.0233508 | -0.0027812 | 0.0296242 |
| ${}_{45}^{-}$^1^ | 0.0448178 | 0.0677602 | -0.0026128 | 0.0646519 |
| ${}_{46}^{-}$^1^ | 0.0083788 | 0.0244335 | -0.0020508 | 0.0306555 |
| ${}_{56}^{-}$^1^ | 0.0051186 | 0.0214348 | -0.0025019 | 0.0282652 |
| ${}_{123}^{-}$^1^ | 0.0397030 | 0.0537100 | -0.0027525 | 0.0517160 |
| ${}_{124}^{-}$^1^ | 0.0364333 | 0.0500165 | -0.0027578 | 0.0483095 |
| ${}_{125}^{-}$^1^ | 0.0260142 | 0.0383834 | -0.0037974 | 0.0359810 |
| ${}_{126}^{-}$^1^ | 0.0062453 | 0.0182549 | -0.0028375 | 0.0231225 |
| ${}_{134}^{-}$^1^ | 0.0356712 | 0.0474235 | -0.0026184 | 0.0463139 |
| ${}_{135}^{-}$^1^ | 0.0251306 | 0.0367225 | -0.0037146 | 0.0349264 |
| ${}_{136}^{-}$^1^ | 0.0063860 | 0.0179651 | -0.0027625 | 0.0230414 |
| ${}_{145}^{-}$^1^ | 0.0258460 | 0.0375261 | -0.0036248 | 0.0358084 |
| ${}_{146}^{-}$^1^ | 0.0062633 | 0.0176238 | -0.0027133 | 0.0226306 |
| ${}_{156}^{-}$^1^ | 0.0033275 | 0.0154868 | -0.0031996 | 0.0211930 |
| ${}_{234}^{-}$^1^ | 0.0441101 | 0.0545899 | -0.0025578 | 0.0533265 |
| ${}_{235}^{-}$^1^ | 0.0253974 | 0.0365593 | -0.0036155 | 0.0344427 |
| ${}_{236}^{-}$^1^ | 0.0054480 | 0.0158115 | -0.0027819 | 0.0201246 |
| ${}_{245}^{-}$^1^ | 0.0298184 | 0.0417184 | -0.0027765 | 0.0397689 |
| ${}_{246}^{-}$^1^ | 0.0061823 | 0.0165729 | -0.0020591 | 0.0208120 |
| ${}_{256}^{-}$^1^ | 0.0036371 | 0.0152661 | -0.0025111 | 0.0202495 |
| ${}_{345}^{-}$^1^ | 0.0268383 | 0.0364227 | -0.0032674 | 0.0350269 |
| ${}_{346}^{-}$^1^ | 0.0050909 | 0.0141251 | -0.0026294 | 0.0182810 |
| ${}_{356}^{-}$^1^ | 0.0028902 | 0.0136829 | -0.0031348 | 0.0188437 |
| ${}_{456}^{-}$^1^ | 0.0037534 | 0.0146138 | -0.0023939 | 0.0196866 |
| ${}_{1234}^{-}$^1^ | 0.0318870 | 0.0428960 | -0.0026714 | 0.0414996 |
| ${}_{1235}^{-}$^1^ | 0.0217656 | 0.0313707 | -0.0037232 | 0.0294257 |
| ${}_{1236}^{-}$^1^ | 0.0054277 | 0.0152852 | -0.0027647 | 0.0194117 |
| ${}_{1245}^{-}$^1^ | 0.0220192 | 0.0314808 | -0.0036273 | 0.0296419 |
| ${}_{1246}^{-}$^1^ | 0.0052532 | 0.0147979 | -0.0027105 | 0.0188179 |
| ${}_{1256}^{-}$^1^ | 0.0025716 | 0.0127019 | -0.0031904 | 0.0171855 |
| ${}_{1345}^{-}$^1^ | 0.0216640 | 0.0308458 | -0.0035523 | 0.0294179 |
| ${}_{1346}^{-}$^1^ | 0.0054383 | 0.0148103 | -0.0026420 | 0.0190410 |
| ${}_{1356}^{-}$^1^ | 0.0029276 | 0.0128776 | -0.0031157 | 0.0176142 |
| ${}_{1456}^{-}$^1^ | 0.0027665 | 0.0123070 | -0.0030543 | 0.0168998 |
| ${}_{2345}^{-}$^1^ | 0.0217635 | 0.0306505 | -0.0034535 | 0.0289533 |
| ${}_{2346}^{-}$^1^ | 0.0046444 | 0.0131045 | -0.0026572 | 0.0167090 |
| ${}_{2356}^{-}$^1^ | 0.0022690 | 0.0114099 | -0.0031258 | 0.0154990 |
| ${}_{2456}^{-}$^1^ | 0.0030345 | 0.0122063 | -0.0023971 | 0.0162392 |
| ${}_{3456}^{-}$^1^ | 0.0024360 | 0.0110388 | -0.0029924 | 0.0152172 |
| ${}_{12345}^{-}$^1^ | 0.0179440 | 0.0244076 | -0.0035543 | 0.0230497 |
| ${}_{12346}^{-}$^1^ | 0.0044721 | 0.0119031 | -0.0026393 | 0.0152187 |
| ${}_{12356}^{-}$^1^ | 0.0022386 | 0.0103158 | -0.0031068 | 0.0140091 |
| ${}_{12456}^{-}$^1^ | 0.0021088 | 0.0098692 | -0.0030456 | 0.0134498 |
| ${}_{13456}^{-}$^1^ | 0.0023978 | 0.0099857 | -0.0029743 | 0.0137630 |
| ${}_{23456}^{-}$^1^ | 0.0018573 | 0.0088419 | -0.0029839 | 0.0121044 |
| ${}_{123456}^{-}$^1^ | 0.0018408 | 0.0080563 | -0.0029657 | 0.0110102 |
| Log-Likelihood | -691.0331 | -691.0331 | -691.0331 | -691.0331 |
| Iterations^2^ | 11 | 11 | 16 | 24 |

lCl: lower confidence limit, uCl: upper confidence limit

^1^ ${}_{ij}^{+}$ is the dependency of the sensitivities of test i and test j; ${}_{ij}^{-}$ is the dependency of the specifities of test i and test j

^2^ The number of the iterations the whole algorithm, not the ones of the EM algorithm performed at each step

^3^Confidence limits could not be calculated
