# Supplementary material for: An intercomparison study of ELISAs for the detection of porcine reproductive and respiratory syndrome virus – evaluating six conditionally dependent tests
Source: PLoS One. 2022 Jan 25;17(1):e0262944. doi: 10.1371/journal.pone.0262944 (PMC8789123; doi:10.1371/journal.pone.0262944)
Supplement: S5 Table — (DOCX) [file pone.0262944.s005.docx]

**S5 Table. Studies evaluating the diagnostic test accuracies of the tests analyzed in this publication that were used as sources of information for suitable starting values for the iterative, frequentist latent class analysis**

| **Source** | **Sample** | **Tests used in the source and in our study** | **Sensitivities** | **Specificities** |
| --- | --- | --- | --- | --- |
| Díaz et al. (2012) | Blood samples from experimentally and naturally infected pigs | PRRS X3 | 100% | - |
| Gerber et al. (2014) | Oral fluid samples from experimentally infected boars | PRRS X3 | 100% | 100% |
| Sipos et al. (2009) | Serum samples collected during routine  diagnostics on pig farms | PRRS X3 | 100%  (assumed gold standard) | 100%  (assumed gold standard) |
|  |  | Ingezim PRRS universal | 82.2-97.3% | 71.1-92.3% |
| Sattler et al. (2016) | Serum samples of piglets vaccinated with an inactivated PRRSV vaccine and subsequent challenge | PRRS X3 | 90% (Type 1 vaccine)  100% (field strain) | - |
|  |  | pigtyp PRRSV Ab | 90% (Type 1 vaccine)  100% (field strain) | - |
|  |  | Ingezim PRRS 2.0 | 100% | - |
| Sattler et al. (2015) | Serum samples of PRRSV positive and negative herds, pigs of monitored PRRSV negative herds, herds with unknown herd history and wild boars | PRRS X3 | 100%  (assumed gold standard) | 100%  (assumed gold standard) |
|  |  | INgezim PRRS 2.0 | 96.0% | 99.0% |
|  |  | PrioCHECK | 100% | 95.1% |
| Sattler et al. (2014) | Serum samples of  pigs after PRRSV type 2 live vaccination  and subsequent challenge | PRRS X3 | - | 100% |
|  |  | pigtyp PRRSV Ab | Higher sensitivity than PRRS X3 | 98,1% |
|  |  | Ingezim PRRS 2.0 | Higher than PRRS X3 | Higher than Ingezim PRRS universal |
| Karniychuk and Nauwynck (2014) | Serum from Belarusian swine farms | PRRS X3 | 80.0% | 83.0% |
|  |  | Ingezim PRRS universal | 83.0% | 77.0% |
| Biernacka et al. (2018) | Serum samples obtained from Polish pig farms | PRRS X3 | 64.8% | 100% |
|  |  | INgezim PRRS 2.0 | 59.3% | 96.6% |
|  |  | Pigtype PRRSV Ab | 63.6% | 98.6% |
|  |  | PrioCHECK | 67.2% | 94.2% |
| Henao-Diaz et al. (2019) | Oral fluid samples with known status collected under experimental conditions | PRRS X3 | 100% | 100% |
|  |  | pigtyp PRRSV Ab | 94.0% | 100% |
