# Supplementary material for: An intercomparison study of ELISAs for the detection of porcine reproductive and respiratory syndrome virus – evaluating six conditionally dependent tests
Source: PLoS One. 2022 Jan 25;17(1):e0262944. doi: 10.1371/journal.pone.0262944 (PMC8789123; doi:10.1371/journal.pone.0262944)
Supplement: S6 Table — (DOCX) [file pone.0262944.s006.docx]

**S6 Table. Starting values for the sensitivity analysis of the stepwise latent class algorithm**

| **Parameter** | **Starting values S1** | **Starting values S2** | **Starting values S3** | **Starting values S4** | **Starting values S5** | **Starting values S6** | **Starting values S7** | **Starting values S8** | **Starting values S9** | **Starting values S10** | **Starting values S11** |
| --- | --- | --- | --- | --- | --- | --- | --- | --- | --- | --- | --- |
| Prevalence | 0.64 | 0.84 | 0.74 | 0.74 | 0.74 | 0.74 | 0.74 | 0.74 | 0.74 | 0.74 | 0.64 |
| Sensitivity 1 | 0.92 | 0.92 | 0.87 | 0.92 | 0.82 | 0.92 | 0.92 | 0.92 | 0.92 | 0.92 | 0.87 |
| Sensitivity 2 | 0.90 | 0.90 | 0.85 | 0.90 | 0.80 | 0.90 | 0.90 | 0.90 | 0.90 | 0.90 | 0.85 |
| Sensitivity 3 | 0.90 | 0.90 | 0.85 | 0.90 | 0.80 | 0.90 | 0.90 | 0.90 | 0.90 | 0.90 | 0.85 |
| Sensitivity 4 | 0.90 | 0.90 | 0.85 | 0.90 | 0.80 | 0.90 | 0.90 | 0.90 | 0.90 | 0.90 | 0.85 |
| Sensitivity 5 | 0.90 | 0.90 | 0.85 | 0.90 | 0.80 | 0.90 | 0.90 | 0.90 | 0.90 | 0.90 | 0.85 |
| Sensitvity 6 | 0.99 | 0.99 | 0.94 | 0.99 | 0.89 | 0.99 | 0.99 | 0.99 | 0.99 | 0.99 | 0.94 |
| Specifity 1 | 0.97 | 0.97 | 0.97 | 0.92 | 0.97 | 0.87 | 0.97 | 0.97 | 0.97 | 0.97 | 0.92 |
| Specifity 2 | 0.97 | 0.97 | 0.97 | 0.92 | 0.97 | 0.87 | 0.97 | 0.97 | 0.97 | 0.97 | 0.92 |
| Specifity 3 | 0.97 | 0.97 | 0.97 | 0.92 | 0.97 | 0.87 | 0.97 | 0.97 | 0.97 | 0.97 | 0.92 |
| Specifity 4 | 0.97 | 0.97 | 0.97 | 0.92 | 0.97 | 0.87 | 0.97 | 0.97 | 0.97 | 0.97 | 0.92 |
| Specifity 5 | 0.97 | 0.97 | 0.97 | 0.92 | 0.97 | 0.87 | 0.97 | 0.97 | 0.97 | 0.97 | 0.92 |
| Specifity 6 | 0.70 | 0.70 | 0.70 | 0.65 | 0.70 | 0.60 | 0.70 | 0.70 | 0.70 | 0.70 | 0.65 |
| ${}_{12}^{+}$^1^ | 0.003 | 0.003 | 0.003 | 0.003 | 0.003 | 0.003 | 0.0045 | 0.003 | 0.006 | 0.003 | 0.0045 |
| ${}_{13}^{+}$^1^ | 0.010 | 0.010 | 0.010 | 0.010 | 0.010 | 0.010 | 0.0150 | 0.010 | 0.020 | 0.010 | 0.0150 |
| ${}_{14}^{+}$^1^ | 0.007 | 0.007 | 0.007 | 0.007 | 0.007 | 0.007 | 0.0105 | 0.007 | 0.014 | 0.007 | 0.0105 |
| ${}_{15}^{+}$^1^ | 0.018 | 0.018 | 0.018 | 0.018 | 0.018 | 0.018 | 0.0370 | 0.018 | 0.036 | 0.018 | 0.0370 |
| ${}_{16}^{+}$^1^ | 0.031 | 0.031 | 0.031 | 0.031 | 0.031 | 0.031 | 0.0465 | 0.031 | 0.062 | 0.031 | 0.0465 |
| ${}_{23}^{+}$^1^ | 0.008 | 0.008 | 0.008 | 0.008 | 0.008 | 0.008 | 0.0120 | 0.008 | 0.016 | 0.008 | 0.0120 |
| ${}_{24}^{+}$^1^ | 0.008 | 0.008 | 0.008 | 0.008 | 0.008 | 0.008 | 0.0120 | 0.008 | 0.016 | 0.008 | 0.0120 |
| ${}_{25}^{+}$^1^ | 0.010 | 0.010 | 0.010 | 0.010 | 0.010 | 0.010 | 0.0150 | 0.010 | 0.020 | 0.010 | 0.0150 |
| ${}_{26}^{+}$^1^ | 0.014 | 0.014 | 0.014 | 0.014 | 0.014 | 0.014 | 0.0210 | 0.014 | 0.028 | 0.014 | 0.0210 |
| ${}_{34}^{+}$^1^ | 0.005 | 0.005 | 0.005 | 0.005 | 0.005 | 0.005 | 0.0075 | 0.005 | 0.010 | 0.005 | 0.0075 |
| ${}_{35}^{+}$^1^ | 0.008 | 0.008 | 0.008 | 0.008 | 0.008 | 0.008 | 0.0120 | 0.008 | 0.016 | 0.008 | 0.0120 |
| ${}_{36}^{+}$^1^ | 0.011 | 0.011 | 0.011 | 0.011 | 0.011 | 0.011 | 0.0165 | 0.011 | 0.022 | 0.011 | 0.0165 |
| ${}_{45}^{+}$^1^ | 0.001 | 0.001 | 0.001 | 0.001 | 0.001 | 0.001 | 0.0015 | 0.001 | 0.002 | 0.001 | 0.0015 |
| ${}_{46}^{+}$^1^ | 0.008 | 0.008 | 0.008 | 0.008 | 0.008 | 0.008 | 0.0120 | 0.008 | 0.016 | 0.008 | 0.0120 |
| ${}_{56}^{+}$^1^ | 0.028 | 0.028 | 0.028 | 0.028 | 0.028 | 0.028 | 0.0420 | 0.028 | 0.056 | 0.028 | 0.0420 |
| ${}_{123}^{+}$^1^ | 0.005 | 0.005 | 0.005 | 0.005 | 0.005 | 0.005 | 0.0075 | 0.005 | 0.010 | 0.005 | 0.0075 |
| ${}_{124}^{+}$^1^ | 0.007 | 0.007 | 0.007 | 0.007 | 0.007 | 0.007 | 0.0105 | 0.007 | 0.014 | 0.007 | 0.0105 |
| ${}_{125}^{+}$^1^ | 0.011 | 0.011 | 0.011 | 0.011 | 0.011 | 0.011 | 0.0165 | 0.011 | 0.022 | 0.011 | 0.0165 |
| ${}_{126}^{+}$^1^ | 0.008 | 0.008 | 0.008 | 0.008 | 0.008 | 0.008 | 0.0120 | 0.008 | 0.016 | 0.008 | 0.0120 |
| ${}_{134}^{+}$^1^ | 0.006 | 0.006 | 0.006 | 0.006 | 0.006 | 0.006 | 0.0090 | 0.006 | 0.012 | 0.006 | 0.0090 |
| ${}_{135}^{+}$^1^ | 0.007 | 0.007 | 0.007 | 0.007 | 0.007 | 0.007 | 0.0105 | 0.007 | 0.014 | 0.007 | 0.0105 |
| ${}_{136}^{+}$^1^ | 0.007 | 0.007 | 0.007 | 0.007 | 0.007 | 0.007 | 0.0105 | 0.007 | 0.014 | 0.007 | 0.0105 |
| ${}_{145}^{+}$^1^ | 0.006 | 0.006 | 0.006 | 0.006 | 0.006 | 0.006 | 0.0090 | 0.006 | 0.012 | 0.006 | 0.0090 |
| ${}_{146}^{+}$^1^ | 0.005 | 0.005 | 0.005 | 0.005 | 0.005 | 0.005 | 0.0075 | 0.005 | 0.010 | 0.005 | 0.0075 |
| ${}_{156}^{+}$^1^ | 0.010 | 0.010 | 0.010 | 0.010 | 0.010 | 0.010 | 0.0150 | 0.010 | 0.020 | 0.010 | 0.0150 |
| ${}_{234}^{+}$^1^ | 0.006 | 0.006 | 0.006 | 0.006 | 0.006 | 0.006 | 0.0090 | 0.006 | 0.012 | 0.006 | 0.0090 |
| ${}_{235}^{+}$^1^ | 0.007 | 0.007 | 0.007 | 0.007 | 0.007 | 0.007 | 0.0105 | 0.007 | 0.014 | 0.007 | 0.0105 |
| ${}_{236}^{+}$^1^ | 0.006 | 0.006 | 0.006 | 0.006 | 0.006 | 0.006 | 0.0090 | 0.006 | 0.012 | 0.006 | 0.0090 |
| ${}_{245}^{+}$^1^ | 0.007 | 0.007 | 0.007 | 0.007 | 0.007 | 0.007 | 0.0105 | 0.007 | 0.014 | 0.007 | 0.0105 |
| ${}_{246}^{+}$^1^ | 0.005 | 0.005 | 0.005 | 0.005 | 0.005 | 0.005 | 0.0075 | 0.005 | 0.010 | 0.005 | 0.0075 |
| ${}_{256}^{+}$^1^ | 0.007 | 0.007 | 0.007 | 0.007 | 0.007 | 0.007 | 0.0105 | 0.007 | 0.014 | 0.007 | 0.0105 |
| ${}_{345}^{+}$^1^ | 0.006 | 0.006 | 0.006 | 0.006 | 0.006 | 0.006 | 0.0090 | 0.006 | 0.012 | 0.006 | 0.0090 |
| ${}_{346}^{+}$^1^ | 0.005 | 0.005 | 0.005 | 0.005 | 0.005 | 0.005 | 0.0075 | 0.005 | 0.010 | 0.005 | 0.0075 |
| ${}_{356}^{+}$^1^ | 0.006 | 0.006 | 0.006 | 0.006 | 0.006 | 0.006 | 0.0090 | 0.006 | 0.012 | 0.006 | 0.0090 |
| ${}_{456}^{+}$^1^ | 0.007 | 0.007 | 0.007 | 0.007 | 0.007 | 0.007 | 0.0105 | 0.007 | 0.014 | 0.007 | 0.0105 |
| ${}_{1234}^{+}$^1^ | 0.006 | 0.006 | 0.006 | 0.006 | 0.006 | 0.006 | 0.0090 | 0.006 | 0.012 | 0.006 | 0.0090 |
| ${}_{1235}^{+}$^1^ | 0.006 | 0.006 | 0.006 | 0.006 | 0.006 | 0.006 | 0.0090 | 0.006 | 0.012 | 0.006 | 0.0090 |
| ${}_{1236}^{+}$^1^ | 0.006 | 0.006 | 0.006 | 0.006 | 0.006 | 0.006 | 0.0090 | 0.006 | 0.012 | 0.006 | 0.0090 |
| ${}_{1245}^{+}$^1^ | 0.006 | 0.006 | 0.006 | 0.006 | 0.006 | 0.006 | 0.0090 | 0.006 | 0.012 | 0.006 | 0.0090 |
| ${}_{1246}^{+}$^1^ | 0.005 | 0.005 | 0.005 | 0.005 | 0.005 | 0.005 | 0.0075 | 0.005 | 0.010 | 0.005 | 0.0075 |
| ${}_{1256}^{+}$^1^ | 0.006 | 0.006 | 0.006 | 0.006 | 0.006 | 0.006 | 0.0090 | 0.006 | 0.012 | 0.006 | 0.0090 |
| ${}_{1345}^{+}$^1^ | 0.006 | 0.006 | 0.006 | 0.006 | 0.006 | 0.006 | 0.0090 | 0.006 | 0.012 | 0.006 | 0.0090 |
| ${}_{1346}^{+}$^1^ | 0.005 | 0.005 | 0.005 | 0.005 | 0.005 | 0.005 | 0.0075 | 0.005 | 0.010 | 0.005 | 0.0075 |
| ${}_{1356}^{+}$^1^ | 0.005 | 0.005 | 0.005 | 0.005 | 0.005 | 0.005 | 0.0075 | 0.005 | 0.010 | 0.005 | 0.0075 |
| ${}_{1456}^{+}$^1^ | 0.005 | 0.005 | 0.005 | 0.005 | 0.005 | 0.005 | 0.0075 | 0.005 | 0.010 | 0.005 | 0.0075 |
| ${}_{2345}^{+}$^1^ | 0.005 | 0.005 | 0.005 | 0.005 | 0.005 | 0.005 | 0.0075 | 0.005 | 0.010 | 0.005 | 0.0075 |
| ${}_{2346}^{+}$^1^ | 0.005 | 0.005 | 0.005 | 0.005 | 0.005 | 0.005 | 0.0075 | 0.005 | 0.010 | 0.005 | 0.0075 |
| ${}_{2356}^{+}$^1^ | 0.005 | 0.005 | 0.005 | 0.005 | 0.005 | 0.005 | 0.0075 | 0.005 | 0.010 | 0.005 | 0.0075 |
| ${}_{2456}^{+}$^1^ | 0.005 | 0.005 | 0.005 | 0.005 | 0.005 | 0.005 | 0.0075 | 0.005 | 0.010 | 0.005 | 0.0075 |
| ${}_{3456}^{+}$^1^ | 0.005 | 0.005 | 0.005 | 0.005 | 0.005 | 0.005 | 0.0075 | 0.005 | 0.010 | 0.005 | 0.0075 |
| ${}_{12345}^{+}$^1^ | 0.005 | 0.005 | 0.005 | 0.005 | 0.005 | 0.005 | 0.0075 | 0.005 | 0.010 | 0.005 | 0.0075 |
| ${}_{12346}^{+}$^1^ | 0.004 | 0.004 | 0.004 | 0.004 | 0.004 | 0.004 | 0.0060 | 0.004 | 0.010 | 0.004 | 0.0060 |
| ${}_{12356}^{+}$^1^ | 0.005 | 0.005 | 0.005 | 0.005 | 0.005 | 0.005 | 0.0075 | 0.005 | 0.010 | 0.005 | 0.0075 |
| ${}_{12456}^{+}$^1^ | 0.004 | 0.004 | 0.004 | 0.004 | 0.004 | 0.004 | 0.0060 | 0.004 | 0.008 | 0.004 | 0.0060 |
| ${}_{13456}^{+}$^1^ | 0.004 | 0.004 | 0.004 | 0.004 | 0.004 | 0.004 | 0.0060 | 0.004 | 0.008 | 0.004 | 0.0060 |
| ${}_{23456}^{+}$^1^ | 0.004 | 0.004 | 0.004 | 0.004 | 0.004 | 0.004 | 0.0060 | 0.004 | 0.008 | 0.004 | 0.0060 |
| ${}_{123456}^{+}$^1^ | 0.004 | 0.004 | 0.004 | 0.004 | 0.004 | 0.004 | 0.0060 | 0.004 | 0.008 | 0.004 | 0.0060 |
| ${}_{12}^{-}$^1^ | 0.070 | 0.070 | 0.070 | 0.070 | 0.070 | 0.070 | 0.070 | 0.1050 | 0.070 | 0.014 | 0.1050 |
| ${}_{13}^{-}$^1^ | 0.050 | 0.050 | 0.050 | 0.050 | 0.050 | 0.050 | 0.050 | 0.0750 | 0.050 | 0.100 | 0.0750 |
| ${}_{14}^{-}$^1^ | 0.065 | 0.065 | 0.065 | 0.065 | 0.065 | 0.065 | 0.065 | 0.0975 | 0.065 | 0.130 | 0.0975 |
| ${}_{15}^{-}$^1^ | 0.050 | 0.050 | 0.050 | 0.050 | 0.050 | 0.050 | 0.050 | 0.0750 | 0.050 | 0.100 | 0.0750 |
| ${}_{16}^{-}$^1^ | 0.029 | 0.029 | 0.029 | 0.029 | 0.029 | 0.029 | 0.029 | 0.0435 | 0.029 | 0.058 | 0.0435 |
| ${}_{23}^{-}$^1^ | 0.100 | 0.100 | 0.100 | 0.100 | 0.100 | 0.100 | 0.100 | 0.1500 | 0.100 | 0.200 | 0.1500 |
| ${}_{24}^{-}$^1^ | 0.099 | 0.099 | 0.099 | 0.099 | 0.099 | 0.099 | 0.099 | 0.1485 | 0.099 | 0.198 | 0.1485 |
| ${}_{25}^{-}$^1^ | 0.058 | 0.058 | 0.058 | 0.058 | 0.058 | 0.058 | 0.058 | 0.0870 | 0.058 | 0.116 | 0.0870 |
| ${}_{26}^{-}$^1^ | 0.028 | 0.028 | 0.028 | 0.028 | 0.028 | 0.028 | 0.028 | 0.0420 | 0.028 | 0.056 | 0.0420 |
| ${}_{34}^{-}$^1^ | 0.122 | 0.122 | 0.122 | 0.122 | 0.122 | 0.122 | 0.122 | 0.1830 | 0.122 | 0.244 | 0.1830 |
| ${}_{35}^{-}$^1^ | 0.056 | 0.056 | 0.056 | 0.056 | 0.056 | 0.056 | 0.056 | 0.0840 | 0.056 | 0.112 | 0.0840 |
| ${}_{36}^{-}$^1^ | 0.034 | 0.034 | 0.034 | 0.034 | 0.034 | 0.034 | 0.034 | 0.0510 | 0.034 | 0.068 | 0.0510 |
| ${}_{45}^{-}$^1^ | 0.067 | 0.067 | 0.067 | 0.067 | 0.067 | 0.067 | 0.067 | 0.1005 | 0.067 | 0.134 | 0.1005 |
| ${}_{46}^{-}$^1^ | 0.030 | 0.030 | 0.030 | 0.030 | 0.030 | 0.030 | 0.030 | 0.0450 | 0.030 | 0.060 | 0.0450 |
| ${}_{56}^{-}$^1^ | 0.020 | 0.020 | 0.020 | 0.020 | 0.020 | 0.020 | 0.020 | 0.0300 | 0.020 | 0.040 | 0.0300 |
| ${}_{123}^{-}$^1^ | 0.055 | 0.055 | 0.055 | 0.055 | 0.055 | 0.055 | 0.055 | 0.0825 | 0.055 | 0.110 | 0.0825 |
| ${}_{124}^{-}$^1^ | 0.045 | 0.045 | 0.045 | 0.045 | 0.045 | 0.045 | 0.045 | 0.0675 | 0.045 | 0.090 | 0.0675 |
| ${}_{125}^{-}$^1^ | 0.033 | 0.033 | 0.033 | 0.033 | 0.033 | 0.033 | 0.033 | 0.0495 | 0.033 | 0.066 | 0.0495 |
| ${}_{126}^{-}$^1^ | 0.020 | 0.020 | 0.020 | 0.020 | 0.020 | 0.020 | 0.020 | 0.0300 | 0.020 | 0.040 | 0.0300 |
| ${}_{134}^{-}$^1^ | 0.041 | 0.041 | 0.041 | 0.041 | 0.041 | 0.041 | 0.041 | 0.0615 | 0.041 | 0.082 | 0.0615 |
| ${}_{135}^{-}$^1^ | 0.031 | 0.031 | 0.031 | 0.031 | 0.031 | 0.031 | 0.031 | 0.0465 | 0.031 | 0.062 | 0.0465 |
| ${}_{136}^{-}$^1^ | 0.020 | 0.020 | 0.020 | 0.020 | 0.020 | 0.020 | 0.020 | 0.0300 | 0.020 | 0.040 | 0.0300 |
| ${}_{145}^{-}$^1^ | 0.025 | 0.025 | 0.025 | 0.025 | 0.025 | 0.025 | 0.025 | 0.0375 | 0.025 | 0.050 | 0.0375 |
| ${}_{146}^{-}$^1^ | 0.019 | 0.019 | 0.019 | 0.019 | 0.019 | 0.019 | 0.019 | 0.0285 | 0.019 | 0.038 | 0.0285 |
| ${}_{156}^{-}$^1^ | 0.017 | 0.017 | 0.017 | 0.017 | 0.017 | 0.017 | 0.017 | 0.0255 | 0.017 | 0.034 | 0.0255 |
| ${}_{234}^{-}$^1^ | 0.053 | 0.053 | 0.053 | 0.053 | 0.053 | 0.053 | 0.053 | 0.0795 | 0.053 | 0.106 | 0.0795 |
| ${}_{235}^{-}$^1^ | 0.033 | 0.033 | 0.033 | 0.033 | 0.033 | 0.033 | 0.033 | 0.0495 | 0.033 | 0.066 | 0.0495 |
| ${}_{236}^{-}$^1^ | 0.017 | 0.017 | 0.017 | 0.017 | 0.017 | 0.017 | 0.017 | 0.0255 | 0.017 | 0.034 | 0.0255 |
| ${}_{245}^{-}$^1^ | 0.035 | 0.035 | 0.035 | 0.035 | 0.035 | 0.035 | 0.035 | 0.0525 | 0.035 | 0.070 | 0.0525 |
| ${}_{246}^{-}$^1^ | 0.017 | 0.017 | 0.017 | 0.017 | 0.017 | 0.017 | 0.017 | 0.0255 | 0.017 | 0.034 | 0.0255 |
| ${}_{256}^{-}$^1^ | 0.015 | 0.015 | 0.015 | 0.015 | 0.015 | 0.015 | 0.015 | 0.0225 | 0.015 | 0.030 | 0.0225 |
| ${}_{345}^{-}$^1^ | 0.032 | 0.032 | 0.032 | 0.032 | 0.032 | 0.032 | 0.032 | 0.0480 | 0.032 | 0.064 | 0.0480 |
| ${}_{346}^{-}$^1^ | 0.018 | 0.018 | 0.018 | 0.018 | 0.018 | 0.018 | 0.018 | 0.0270 | 0.018 | 0.036 | 0.0270 |
| ${}_{356}^{-}$^1^ | 0.013 | 0.013 | 0.013 | 0.013 | 0.013 | 0.013 | 0.013 | 0.0195 | 0.013 | 0.026 | 0.0195 |
| ${}_{456}^{-}$^1^ | 0.013 | 0.013 | 0.013 | 0.013 | 0.013 | 0.013 | 0.013 | 0.0195 | 0.013 | 0.026 | 0.0195 |
| ${}_{1234}^{-}$^1^ | 0.036 | 0.036 | 0.036 | 0.036 | 0.036 | 0.036 | 0.036 | 0.0540 | 0.036 | 0.072 | 0.0540 |
| ${}_{1235}^{-}$^1^ | 0.026 | 0.026 | 0.026 | 0.026 | 0.026 | 0.026 | 0.026 | 0.0390 | 0.026 | 0.052 | 0.0390 |
| ${}_{1236}^{-}$^1^ | 0.017 | 0.017 | 0.017 | 0.017 | 0.017 | 0.017 | 0.017 | 0.0255 | 0.017 | 0.036 | 0.0255 |
| ${}_{1245}^{-}$^1^ | 0.026 | 0.026 | 0.026 | 0.026 | 0.026 | 0.026 | 0.026 | 0.0390 | 0.026 | 0.052 | 0.0390 |
| ${}_{1246}^{-}$^1^ | 0.016 | 0.016 | 0.016 | 0.016 | 0.016 | 0.016 | 0.016 | 0.0240 | 0.016 | 0.032 | 0.0240 |
| ${}_{1256}^{-}$^1^ | 0.012 | 0.012 | 0.012 | 0.012 | 0.012 | 0.012 | 0.012 | 0.0180 | 0.012 | 0.024 | 0.0180 |
| ${}_{1345}^{-}$^1^ | 0.026 | 0.026 | 0.026 | 0.026 | 0.026 | 0.026 | 0.026 | 0.0390 | 0.026 | 0.052 | 0.0390 |
| ${}_{1346}^{-}$^1^ | 0.016 | 0.016 | 0.016 | 0.016 | 0.016 | 0.016 | 0.016 | 0.0240 | 0.016 | 0.032 | 0.0240 |
| ${}_{1356}^{-}$^1^ | 0.014 | 0.014 | 0.014 | 0.014 | 0.014 | 0.014 | 0.014 | 0.0210 | 0.014 | 0.028 | 0.0210 |
| ${}_{1456}^{-}$^1^ | 0.013 | 0.013 | 0.013 | 0.013 | 0.013 | 0.013 | 0.013 | 0.0195 | 0.013 | 0.026 | 0.0195 |
| ${}_{2345}^{-}$^1^ | 0.027 | 0.027 | 0.027 | 0.027 | 0.027 | 0.027 | 0.027 | 0.0405 | 0.027 | 0.054 | 0.0405 |
| ${}_{2346}^{-}$^1^ | 0.013 | 0.013 | 0.013 | 0.013 | 0.013 | 0.013 | 0.013 | 0.0195 | 0.013 | 0.026 | 0.0195 |
| ${}_{2356}^{-}$^1^ | 0.012 | 0.012 | 0.012 | 0.012 | 0.012 | 0.012 | 0.012 | 0.0180 | 0.012 | 0.024 | 0.0180 |
| ${}_{2456}^{-}$^1^ | 0.012 | 0.012 | 0.012 | 0.012 | 0.012 | 0.012 | 0.012 | 0.0180 | 0.012 | 0.024 | 0.0180 |
| ${}_{3456}^{-}$^1^ | 0.015 | 0.015 | 0.015 | 0.015 | 0.015 | 0.015 | 0.015 | 0.0225 | 0.015 | 0.030 | 0.0225 |
| ${}_{12345}^{-}$^1^ | 0.020 | 0.020 | 0.020 | 0.020 | 0.020 | 0.020 | 0.020 | 0.0309 | 0.020 | 0.040 | 0.0309 |
| ${}_{12346}^{-}$^1^ | 0.013 | 0.013 | 0.013 | 0.013 | 0.013 | 0.013 | 0.013 | 0.0195 | 0.013 | 0.026 | 0.0195 |
| ${}_{12356}^{-}$^1^ | 0.011 | 0.011 | 0.011 | 0.011 | 0.011 | 0.011 | 0.011 | 0.0165 | 0.011 | 0.022 | 0.0165 |
| ${}_{12456}^{-}$^1^ | 0.010 | 0.010 | 0.010 | 0.010 | 0.010 | 0.010 | 0.010 | 0.0150 | 0.010 | 0.020 | 0.0150 |
| ${}_{13456}^{-}$^1^ | 0.010 | 0.010 | 0.010 | 0.010 | 0.010 | 0.010 | 0.010 | 0.0150 | 0.010 | 0.020 | 0.0150 |
| ${}_{23456}^{-}$^1^ | 0.009 | 0.009 | 0.009 | 0.009 | 0.009 | 0.009 | 0.009 | 0.0135 | 0.009 | 0.018 | 0.0135 |
| ${}_{123456}^{-}$^1^ | 0.008 | 0.008 | 0.008 | 0.008 | 0.008 | 0.008 | 0.008 | 0.0120 | 0.008 | 0.016 | 0.0120 |

^1^ ${}_{ij}^{+}$ is the dependency of the sensitivities of test i and test j; ${}_{ij}^{-}$ is the dependency of the specifities of test i and test j
