# Supplementary material for: An intercomparison study of ELISAs for the detection of porcine reproductive and respiratory syndrome virus – evaluating six conditionally dependent tests
Source: PLoS One. 2022 Jan 25;17(1):e0262944. doi: 10.1371/journal.pone.0262944 (PMC8789123; doi:10.1371/journal.pone.0262944)
Supplement: S7 Table — (DOCX) [file pone.0262944.s007.docx]

**S7 Table. Resulting values of the stepwise latent class algorithm for the for the sensitivity analysis**

| **Parameter** | **Starting values S1** | **Starting values S2** | **Starting values S3** | **Starting values S4** | **Starting values S5** | **Starting values S6** | **Starting values S7** | **Starting values S8** | **Starting values S9** | **Starting values S10** | **Starting values S11** |
| --- | --- | --- | --- | --- | --- | --- | --- | --- | --- | --- | --- |
| Prevalence  lCl  uCl | 0.7645354  0.7352069 0.7938639 | 0.7644293  0.7350963 0.7937624 | 0.7665106  0.7372677  0.7957535 | 0.7665282  0.7372861 0.7957704 | 0.7646149  0.7352898 0.7939399 | 0.7645649  0.7352377 0.7938921 | 0.5494579  0.5150655 0.5838503 | 0.0000000  -^3^  -^3^ | 0.0000000  -^3^  -^3^ | 0.5318298  0.4973380 0.5663216 | 0.0000000  -^3^  -^3^ |
| Sensitivity 1  lCl  uCl | 0.8786091  0.8560346 0.9011837 | 0.8787051  0.8561382 0.9012719 | 0.8768190  0.8541018  0.8995362 | 0.8768034  0.8540850 0.8995219 | 0.8785372  0.8559569 0.9011175 | 0.8785825  0.8560058 0.9011591 | 0.9965987  0.9925742 1.0006232 | 0.6828356  0.6506673 0.7150039 | 0.6828358  0.6506675 0.7150041 | 0.9973972  0.9938752 1.0009191 | 0.6828358  0.6506675 0.7150041 |
| Sensitivity 2  lCl  uCl | 0.8328582  0.8070680 0.8586485 | 0.8329572  0.8071730 0.8587414 | 0.8310139  0.8051104  0.8569173 | 0.8309974  0.8050930 0.8569019 | 0.8327841  0.8069892 0.8585789 | 0.8328307  0.8070387 0.8586227 | 0.9880367  0.9805215 0.9955519 | 0.6442784  0.6111866 0.6773702 | 0.6442786  0.6111868 0.6773704 | 0.9888264  0.9815606 0.9960922 | 0.6442786  0.6111868 0.6773704 |
| Sensitivity 3  lCl  uCl | 0.7864710  0.7581442 0.8147978 | 0.7865687  0.7582466 0.8148908 | 0.7846512  0.7562368  0.8130655 | 0.7846351  0.7562200 0.8130502 | 0.7863978  0.7580674 0.8147281 | 0.7864438  0.7581157 0.8147720 | 0.9758256  0.9652089 0.9864424 | 0.6057212  0.5719407 0.6395017 | 0.6057214  0.5719409 0.6395019 | 0.9777179  0.9675153 0.9879206 | 0.6057214  0.5719409 0.6395019 |
| Sensitivity 4  lCl  uCl | 0.7469476  0.7168953 0.7770000 | 0.7470431  0.7169945 0.7770917 | 0.7451709  0.7150492  0.7752927 | 0.7451552  0.7150328 0.7752776 | 0.7468761  0.7168210 0.7769313 | 0.7469211  0.7168677 0.7769745 | 0.9432399  0.9272458 0.9592340 | 0.5733829  0.5391952 0.6075706 | 0.5733831  0.5391954 0.6075708 | 0.9475120  0.9320967 0.9629272 | 0.5733831  0.5391954 0.6075707 |
| Sensitivity 5  lCl  uCl | 0.9063996  0.8862657 0.9265334 | 0.9064780  0.8863517 0.9266043 | 0.9049363  0.8846621  0.9252106 | 0.9049226  0.8846470 0.9251981 | 0.9063408  0.8862013 0.9264803 | 0.9063777  0.8862418 0.9265137 | 0.9973612  0.9938151 1.0009074 | 0.7189053  0.6878318 0.7499788 | 0.7189055  0.6878320 0.7499790 | 0.9978491  0.9946467 1.0010515 | 0.7189055  0.6878320 0.7499790 |
| Sensitvity 6  lCl  uCl | 0.9508354  0.9358900 0.9657808 | 0.9508865  0.9359485 0.9658245 | 0.9498729  0.9347896  0.9649562 | 0.9498656  0.9347812 0.9649499 | 0.9507971  0.9358461 0.9657480 | 0.9508212  0.9358737 0.9657686 | 0.9953192  0.9906011 1.0000373 | 0.7748754  0.7460048 0.8037460 | 0.7748756  0.7460051 0.8037462 | 0.9965150  0.9924414 1.0005885 | 0.7748756  0.7460050 0.8037462 |
| Specifity 1  lCl  uCl | 0.9528250  0.9381699 0.9674802 | 0.9527620  0.9380976 0.9674265 | 0.9539817  0.9394985  0.9684648 | 0.9539933  0.9395119 0.9684748 | 0.9528722  0.9382240 0.9675204 | 0.9528425  0.9381900 0.9674951 | 0.6998132  0.6681311 0.7314954 | 1.0071200  -^3^  -^3^ | 1.0100218  -^3^  -^3^ | 0.6744981  0.6421093 0.7068870 | 1.0627499  -^3^  -^3^ |
| Specifity 2  lCl  uCl | 0.9680249  0.9558637 0.9801861 | 0.9679856  0.9558171 0.9801540 | 0.9687452  0.9567173  0.9807731 | 0.9687515  0.9567248 0.9807783 | 0.9680544  0.9558986 0.9802102 | 0.9680359  0.9558767 0.9801951 | 0.7749510  0.7460839 0.8038182 | 0.9652270  0.9525632 0.9778908 | 0.9485624  0.9332937 0.9638311 | 0.7471192  0.7170736 0.7771648 | 0.8873231  0.8654663 0.9091799 |
| Specifity 3  lCl  uCl | 0.9811587  0.9717603 0.9905571 | 0.9811302  0.9717248 0.9905355 | 0.9816782  0.9724079  0.9909486 | 0.9816832  0.9724141 0.9909524 | 0.9811800  0.9717868 0.9905732 | 0.9811666  0.9717701 0.9905630 | 0.8456387  0.8206646 0.8706128 | 1.0020025  -^3^  -^3^ | 1.0076844  -^3^  -^3^ | 0.8168575  0.7901215 0.8435935 | 1.0313638  -^3^  -^3^ |
| Specifity 4  lCl  uCl | 0.9901676  0.9833472 0.9969881 | 0.9901455  0.9833175 0.9969735 | 0.9905706  0.9838901  0.9972512 | 0.9905743  0.9838951 0.9972536 | 0.9901842  0.9833695 0.9969989 | 0.9901738  0.9833555 0.9969921 | 0.8776752  0.8550260 0.9003244 | 0.9994879  0.9979240 1.0010518 | 1.0000000  1.0000000 1.0000000 | 0.8516181  0.8270460 0.8761901 | 1.0008732  -^3^  -^3^ |
| Specifity 5  lCl  uCl | 0.8898733  0.8682343 0.9115124 | 0.8897693  0.8681213 0.9114174 | 0.8918059  0.8703343  0.9132775 | 0.8918208  0.8703505 0.9132911 | 0.8899513  0.8683189 0.9115836 | 0.8899023  0.8682657 0.9115388 | 0.6206848  0.5871448 0.6542249 | 0.8993053  0.8785042 0.9201063 | 0.9426069  0.9265293 0.9586846 | 0.5979676  0.5640756 0.6318596 | 0.8081335  0.7809146 0.8353523 |
| Specifity 6  lCl  uCl | 0.7964522  0.7686204 0.8242840 | 0.7962817  0.7684412 0.8241222 | 0.7996141  0.7719446  0.8272836 | 0.7996466  0.7719787 0.8273144 | 0.7965800  0.7687547 0.8244053 | 0.7964996  0.7686702 0.8243290 | 0.4939660  0.4594066 0.5285255 | 0.8895390  0.8678712 0.9112068 | 0.7989562  0.7712527 0.8266597 | 0.4769012  0.4423762 0.5114262 | 0.7028674  0.6712781 0.7344567 |
| ${}_{12}^{+}$^1^ | 0.0162313 | 0.0162579 | 0.0157455 | 0.0157409 | 0.0162114 | 0.0162239 | 0.1297065 | 0.0009987 | 0.0005454 | 0.1431142 | 0.0022895 |
| ${}_{13}^{+}$^1^ | 0.0134861 | 0.0135077 | 0.0130916 | 0.0130880 | 0.0134699 | 0.0134801 | 0.0976274 | -0.0002692 | -0.0014384 | 0.1133557 | -0.0081798 |
| ${}_{14}^{+}$^1^ | 0.0074149 | 0.0074303 | 0.0071344 | 0.0071319 | 0.0074034 | 0.0074107 | 0.0639304 | 0.0003526 | 0.0002971 | 0.0783746 | 0.0003668 |
| ${}_{15}^{+}$^1^ | 0.0201351 | 0.0201632 | 0.0196218 | 0.0196156 | 0.0201140 | 0.0201273 | 0.1283828 | -0.0027947 | -0.0011828 | 0.1382891 | -0.0142573 |
| ${}_{16}^{+}$^1^ | 0.0295718 | 0.0296046 | 0.0289669 | 0.0289615 | 0.0295473 | 0.0295627 | 0.1258950 | -0.0015647 | -0.0068837 | 0.1322176 | -0.0201078 |
| ${}_{23}^{+}$^1^ | 0.0093780 | 0.0093956 | 0.0090576 | 0.0090548 | 0.0093649 | 0.0093732 | 0.0876400 | 0.0004498 | 0.0003084 | 0.1048646 | 0.0030427 |
| ${}_{24}^{+}$^1^ | 0.0076062 | 0.0076223 | 0.0073131 | 0.0073105 | 0.0075942 | 0.0076017 | 0.0707124 | 0.0003613 | 0.0003113 | 0.0865488 | 0.0003551 |
| ${}_{25}^{+}$^1^ | 0.0140861 | 0.0141085 | 0.0136783 | 0.0136736 | 0.0140693 | 0.0140798 | 0.1064045 | -0.0017874 | -0.0010826 | 0.1186083 | -0.0080123 |
| ${}_{26}^{+}$^1^ | 0.0136620 | 0.0136814 | 0.0133230 | 0.0133196 | 0.0136489 | 0.0136576 | 0.0887476 | -0.0018481 | -0.0062988 | 0.0975653 | -0.0125719 |
| ${}_{34}^{+}$^1^ | 0.0069201 | 0.0069345 | 0.0066578 | 0.0066555 | 0.0069093 | 0.0069161 | 0.0649811 | 0.0003050 | 0.0002606 | 0.0818874 | 0.0002839 |
| ${}_{35}^{+}$^1^ | 0.0082592 | 0.0082740 | 0.0079892 | 0.0079864 | 0.0082480 | 0.0082550 | 0.0706942 | 0.0004759 | 0.0001222 | 0.0847868 | 0.0025191 |
| ${}_{36}^{+}$^1^ | 0.0125932 | 0.0126104 | 0.0122782 | 0.0122751 | 0.0125803 | 0.0125884 | 0.0717890 | -0.0010177 | -0.0052019 | 0.0821500 | -0.0138516 |
| ${}_{45}^{+}$^1^ | 0.0087496 | 0.0087682 | 0.0084092 | 0.0084060 | 0.0087356 | 0.0087444 | 0.0759252 | 0.0004588 | 0.0003210 | 0.0887276 | -0.0007009 |
| ${}_{46}^{+}$^1^ | 0.0073210 | 0.0073355 | 0.0070542 | 0.0070517 | 0.0073100 | 0.0073169 | 0.0543139 | 0.0004277 | 0.0002682 | 0.0635389 | -0.0006459 |
| ${}_{56}^{+}$^1^ | 0.0301853 | 0.0302169 | 0.0296024 | 0.0295961 | 0.0301616 | 0.0301765 | 0.1207575 | -0.0037333 | -0.0030414 | 0.1261726 | -0.0214959 |
| ${}_{123}^{+}$^1^ | 0.0087726 | 0.0087881 | 0.0084894 | 0.0084869 | 0.0087610 | 0.0087683 | 0.0436499 | 0.0004050 | 0.0004279 | 0.0470934 | 0.0013714 |
| ${}_{124}^{+}$^1^ | 0.0066251 | 0.0066383 | 0.0063846 | 0.0063825 | 0.0066153 | 0.0066215 | 0.0298351 | 0.0003192 | 0.0002663 | 0.0336423 | 0.0002481 |
| ${}_{125}^{+}$^1^ | 0.0109194 | 0.0109362 | 0.0106119 | 0.0106088 | 0.0109068 | 0.0109147 | 0.0393413 | 0.0006684 | 0.0007005 | 0.0392247 | 0.0009927 |
| ${}_{126}^{+}$^1^ | 0.0100226 | 0.0100358 | 0.0097810 | 0.0097789 | 0.0100128 | 0.0100190 | 0.0283237 | 0.0006527 | 0.0008796 | 0.0272463 | 0.0011023 |
| ${}_{134}^{+}$^1^ | 0.0063444 | 0.0063570 | 0.0061153 | 0.0061134 | 0.0063350 | 0.0063409 | 0.0346738 | 0.0002970 | 0.0002504 | 0.0401222 | 0.0002088 |
| ${}_{135}^{+}$^1^ | 0.0072323 | 0.0072446 | 0.0070074 | 0.0070053 | 0.0072231 | 0.0072289 | 0.0286945 | 0.0004042 | 0.0004231 | 0.0310708 | 0.0016281 |
| ${}_{136}^{+}$^1^ | 0.0078847 | 0.0078961 | 0.0076745 | 0.0076726 | 0.0078761 | 0.0078815 | 0.0256699 | 0.0005567 | 0.0009500 | 0.0263436 | 0.0035693 |
| ${}_{145}^{+}$^1^ | 0.0064004 | 0.0064126 | 0.0061775 | 0.0061755 | 0.0063912 | 0.0063970 | 0.0239762 | 0.0003186 | 0.0002805 | 0.0263459 | 0.0002847 |
| ${}_{146}^{+}$^1^ | 0.0051289 | 0.0051377 | 0.0049683 | 0.0049668 | 0.0051223 | 0.0051265 | 0.0119032 | 0.0002883 | 0.0002223 | 0.0128852 | 0.0002144 |
| ${}_{156}^{+}$^1^ | 0.0097498 | 0.0097597 | 0.0095682 | 0.0095662 | 0.0097424 | 0.0097470 | 0.0160225 | 0.0013586 | 0.0014371 | 0.0137682 | 0.0064706 |
| ${}_{234}^{+}$^1^ | 0.0062025 | 0.0062148 | 0.0059763 | 0.0059744 | 0.0061932 | 0.0061990 | 0.0348076 | 0.0002722 | 0.0002289 | 0.0407960 | 0.0001796 |
| ${}_{235}^{+}$^1^ | 0.0071561 | 0.0071687 | 0.0069255 | 0.0069234 | 0.0071466 | 0.0071526 | 0.0321768 | 0.0002903 | 0.0002878 | 0.0356864 | -0.0002719 |
| ${}_{236}^{+}$^1^ | 0.0071327 | 0.0071441 | 0.0069240 | 0.0069221 | 0.0071242 | 0.0071295 | 0.0288865 | 0.0003787 | 0.0005562 | 0.0309129 | 0.0015182 |
| ${}_{245}^{+}$^1^ | 0.0066300 | 0.0066430 | 0.0063929 | 0.0063908 | 0.0066203 | 0.0066264 | 0.0308742 | 0.0003263 | 0.0002899 | 0.0341540 | 0.0002879 |
| ${}_{246}^{+}$^1^ | 0.0054299 | 0.0054396 | 0.0052516 | 0.0052500 | 0.0054226 | 0.0054272 | 0.0193382 | 0.0002976 | 0.0002305 | 0.0214006 | 0.0002239 |
| ${}_{256}^{+}$^1^ | 0.0071813 | 0.0071906 | 0.0070095 | 0.0070079 | 0.0071743 | 0.0071787 | 0.0169963 | 0.0008429 | 0.0009261 | 0.0162022 | 0.0032763 |
| ${}_{345}^{+}$^1^ | 0.0060768 | 0.0060885 | 0.0058622 | 0.0058602 | 0.0060680 | 0.0060735 | 0.0316852 | 0.0002741 | 0.0002460 | 0.0363852 | 0.0002316 |
| ${}_{346}^{+}$^1^ | 0.0052411 | 0.0052505 | 0.0050682 | 0.0050666 | 0.0052340 | 0.0052385 | 0.0225507 | 0.0002696 | 0.0002122 | 0.0256443 | 0.0002038 |
| ${}_{356}^{+}$^1^ | 0.0061188 | 0.0061279 | 0.0059539 | 0.0059521 | 0.0061121 | 0.0061163 | 0.0162627 | 0.0003682 | 0.0004361 | 0.0168728 | 0.0006808 |
| ${}_{456}^{+}$^1^ | 0.0062180 | 0.0062292 | 0.0060119 | 0.0060099 | 0.0062095 | 0.0062148 | 0.0189401 | 0.0003876 | 0.0002371 | 0.0192725 | -0.0005278 |
| ${}_{1234}^{+}$^1^ | 0.0059251 | 0.0059365 | 0.0057154 | 0.0057136 | 0.0059165 | 0.0059219 | 0.0276019 | 0.0002739 | 0.0002284 | 0.0322383 | 0.0001947 |
| ${}_{1235}^{+}$^1^ | 0.0068738 | 0.0068856 | 0.0066594 | 0.0066574 | 0.0068651 | 0.0068706 | 0.0281555 | 0.0002983 | 0.0002699 | 0.0317383 | 0.0000131 |
| ${}_{1236}^{+}$^1^ | 0.0069664 | 0.0069772 | 0.0067686 | 0.0067669 | 0.0069583 | 0.0069634 | 0.0265857 | 0.0003299 | 0.0002401 | 0.0291398 | -0.0001051 |
| ${}_{1245}^{+}$^1^ | 0.0059302 | 0.0059413 | 0.0057262 | 0.0057244 | 0.0059218 | 0.0059271 | 0.0235538 | 0.0002872 | 0.0002515 | 0.0269950 | 0.0002013 |
| ${}_{1246}^{+}$^1^ | 0.0048347 | 0.0048430 | 0.0046829 | 0.0046815 | 0.0048285 | 0.0048324 | 0.0157965 | 0.0002588 | 0.0001971 | 0.0184877 | 0.0001512 |
| ${}_{1256}^{+}$^1^ | 0.0068037 | 0.0068127 | 0.0066381 | 0.0066365 | 0.0067969 | 0.0068011 | 0.0238380 | 0.0003645 | 0.0003260 | 0.0256447 | 0.0000011 |
| ${}_{1345}^{+}$^1^ | 0.0056926 | 0.0057033 | 0.0054977 | 0.0054960 | 0.0056846 | 0.0056897 | 0.0254239 | 0.0002668 | 0.0002361 | 0.0294392 | 0.0001763 |
| ${}_{1346}^{+}$^1^ | 0.0049414 | 0.0049501 | 0.0047829 | 0.0047815 | 0.0049350 | 0.0049390 | 0.0193505 | 0.0002615 | 0.0002023 | 0.0224251 | 0.0001708 |
| ${}_{1356}^{+}$^1^ | 0.0059830 | 0.0059920 | 0.0058184 | 0.0058168 | 0.0059763 | 0.0059805 | 0.0214776 | 0.0002835 | 0.0002227 | 0.0240679 | 0.0002714 |
| ${}_{1456}^{+}$^1^ | 0.0047269 | 0.0047347 | 0.0045842 | 0.0045828 | 0.0047211 | 0.0047248 | 0.0149868 | 0.0002579 | 0.0002081 | 0.0173933 | 0.0001932 |
| ${}_{2345}^{+}$^1^ | 0.0055311 | 0.0055415 | 0.0053405 | 0.0053389 | 0.0055233 | 0.0055282 | 0.0243226 | 0.0002449 | 0.0002162 | 0.0284039 | 0.0001440 |
| ${}_{2346}^{+}$^1^ | 0.0048014 | 0.0048098 | 0.0046463 | 0.0046450 | 0.0047951 | 0.0047990 | 0.0183620 | 0.0002400 | 0.0001854 | 0.0213791 | 0.0001401 |
| ${}_{2356}^{+}$^1^ | 0.0056330 | 0.0056417 | 0.0054738 | 0.0054722 | 0.0056265 | 0.0056306 | 0.0203269 | 0.0002620 | 0.0002145 | 0.0230061 | 0.0000998 |
| ${}_{2456}^{+}$^1^ | 0.0048808 | 0.0048891 | 0.0047293 | 0.0047279 | 0.0048747 | 0.0048785 | 0.0166620 | 0.0002664 | 0.0002168 | 0.0190495 | 0.0001912 |
| ${}_{3456}^{+}$^1^ | 0.0047139 | 0.0047219 | 0.0045669 | 0.0045656 | 0.0047079 | 0.0047117 | 0.0174581 | 0.0002418 | 0.0001991 | 0.0201198 | 0.0007558 |
| ${}_{12345}^{+}$^1^ | 0.0052817 | 0.0052913 | 0.0051056 | 0.0051041 | 0.0052746 | 0.0052791 | 0.0170021 | 0.0002464 | 0.0002157 | 0.0187465 | 0.0001574 |
| ${}_{12346}^{+}$^1^ | 0.0045840 | 0.0045918 | 0.0044411 | 0.0044399 | 0.0045782 | 0.0045819 | 0.0126043 | 0.0002415 | 0.0001851 | 0.0137692 | 0.0001508 |
| ${}_{12356}^{+}$^1^ | 0.0053746 | 0.0053825 | 0.0052291 | 0.0052277 | 0.0053687 | 0.0053724 | 0.0124882 | 0.0002659 | 0.0002245 | 0.0129178 | 0.0002056 |
| ${}_{12456}^{+}$^1^ | 0.0043231 | 0.0043300 | 0.0041959 | 0.0041947 | 0.0043179 | 0.0043211 | 0.0090391 | 0.0002329 | 0.0001862 | 0.0096674 | 0.0001221 |
| ${}_{13456}^{+}$^1^ | 0.0044074 | 0.0044146 | 0.0042751 | 0.0042739 | 0.0044020 | 0.0044054 | 0.0113323 | 0.0002354 | 0.0001913 | 0.0121757 | 0.0001364 |
| ${}_{23456}^{+}$^1^ | 0.0042796 | 0.0042867 | 0.0041502 | 0.0041491 | 0.0042744 | 0.0042777 | 0.0111038 | 0.0002159 | 0.0001752 | 0.0121132 | 0.0001130 |
| ${}_{123456}^{+}$^1^ | 0.0040873 | 0.0040938 | 0.0039682 | 0.0039672 | 0.0040825 | 0.0040855 | 0.0083142 | 0.0002172 | 0.0001748 | 0.0089023 | 0.0001229 |
| ${}_{12}^{-}$^1^ | 0.0875903 | 0.0875253 | 0.0888022 | 0.0888127 | 0.0876391 | 0.0876084 | 0.0012441 | 0.1906606 | 0.1906605 | 0.0010155 | 0.1906605 |
| ${}_{13}^{-}$^1^ | 0.0903386 | 0.0902763 | 0.0914999 | 0.0915098 | 0.0903853 | 0.0903559 | 0.0027895 | 0.1871381 | 0.1871380 | 0.0021459 | 0.1871380 |
| ${}_{14}^{-}$^1^ | 0.0766327 | 0.0765725 | 0.0777539 | 0.0777636 | 0.0766778 | 0.0766494 | 0.0006076 | 0.1706626 | 0.1706625 | 0.0005283 | 0.1706626 |
| ${}_{15}^{-}$^1^ | 0.0531754 | 0.0531216 | 0.0541794 | 0.0541890 | 0.0532158 | 0.0531903 | 0.0003377 | 0.1645783 | 0.1645782 | 0.0002997 | 0.1645782 |
| ${}_{16}^{-}$^1^ | 0.0293921 | 0.0293540 | 0.0301110 | 0.0301160 | 0.0294207 | 0.0294028 | 0.0003852 | 0.1412853 | 0.1412852 | 0.0003490 | 0.1412852 |
| ${}_{23}^{-}$^1^ | 0.1081516 | 0.1080909 | 0.1092803 | 0.1092902 | 0.1081971 | 0.1081685 | 0.0016803 | 0.1955676 | 0.1955676 | 0.0016443 | 0.1955676 |
| ${}_{24}^{-}$^1^ | 0.1042860 | 0.1042269 | 0.1053840 | 0.1053937 | 0.1043302 | 0.1043024 | 0.0016046 | 0.1877955 | 0.1877955 | 0.0015845 | 0.1877955 |
| ${}_{25}^{-}$^1^ | 0.0612319 | 0.0611784 | 0.0622287 | 0.0622385 | 0.0612721 | 0.0612468 | 0.0004685 | 0.1649341 | 0.1649340 | 0.0004273 | 0.1649340 |
| ${}_{26}^{-}$^1^ | 0.0283141 | 0.0282785 | 0.0289843 | 0.0289893 | 0.0283408 | 0.0283240 | 0.0003713 | 0.1326051 | 0.1326050 | 0.0003360 | 0.1326050 |
| ${}_{34}^{-}$^1^ | 0.1261711 | 0.1261164 | 0.1271876 | 0.1271965 | 0.1262121 | 0.1261863 | 0.0068030 | 0.1999533 | 0.1999533 | 0.0066132 | 0.1999533 |
| ${}_{35}^{-}$^1^ | 0.0599657 | 0.0599151 | 0.0609089 | 0.0609179 | 0.0600036 | 0.0599798 | 0.0005326 | 0.1578272 | 0.1578272 | 0.0004829 | 0.1578272 |
| ${}_{36}^{-}$^1^ | 0.0345292 | 0.0344942 | 0.0351869 | 0.0351918 | 0.0345554 | 0.0345389 | 0.0014340 | 0.1326314 | 0.1326313 | 0.0009609 | 0.1326313 |
| ${}_{45}^{-}$^1^ | 0.0699146 | 0.0698650 | 0.0708387 | 0.0708474 | 0.0699518 | 0.0699284 | 0.0024890 | 0.1611749 | 0.1611748 | 0.0020380 | 0.1611748 |
| ${}_{46}^{-}$^1^ | 0.0303731 | 0.0303392 | 0.0310106 | 0.0310154 | 0.0303985 | 0.0303825 | 0.0003709 | 0.1241075 | 0.1241074 | 0.0003073 | 0.1241074 |
| ${}_{56}^{-}$^1^ | 0.0199817 | 0.0199537 | 0.0205087 | 0.0205133 | 0.0200026 | 0.0199894 | 0.0004332 | 0.1295049 | 0.1295048 | 0.0003819 | 0.1295048 |
| ${}_{123}^{-}$^1^ | 0.0514846 | 0.0514602 | 0.0519342 | 0.0519380 | 0.0515028 | 0.0514913 | 0.0006851 | 0.0502547 | 0.0502548 | 0.0005941 | 0.0502548 |
| ${}_{124}^{-}$^1^ | 0.0474184 | 0.0473943 | 0.0478644 | 0.0478682 | 0.0474365 | 0.0474251 | 0.0005507 | 0.0475225 | 0.0475226 | 0.0004836 | 0.0475226 |
| ${}_{125}^{-}$^1^ | 0.0349409 | 0.0349148 | 0.0354241 | 0.0354292 | 0.0349605 | 0.0349482 | 0.0003164 | 0.0525937 | 0.0525938 | 0.0002843 | 0.0525938 |
| ${}_{126}^{-}$^1^ | 0.0196866 | 0.0196659 | 0.0200752 | 0.0200778 | 0.0197021 | 0.0196923 | 0.0003834 | 0.0483182 | 0.0483182 | 0.0003465 | 0.0483182 |
| ${}_{134}^{-}$^1^ | 0.0426998 | 0.0426792 | 0.0430798 | 0.0430829 | 0.0427152 | 0.0427055 | 0.0005265 | 0.0383470 | 0.0383471 | 0.0004648 | 0.0383471 |
| ${}_{135}^{-}$^1^ | 0.0329130 | 0.0328884 | 0.0333680 | 0.0333725 | 0.0329314 | 0.0329198 | 0.0003052 | 0.0485423 | 0.0485424 | 0.0002741 | 0.0485424 |
| ${}_{136}^{-}$^1^ | 0.0191768 | 0.0191569 | 0.0195506 | 0.0195530 | 0.0191918 | 0.0191824 | 0.0003735 | 0.0446336 | 0.0446336 | 0.0003401 | 0.0446336 |
| ${}_{145}^{-}$^1^ | 0.0325464 | 0.0325229 | 0.0329813 | 0.0329857 | 0.0325640 | 0.0325529 | 0.0003169 | 0.0463940 | 0.0463941 | 0.0002828 | 0.0463941 |
| ${}_{146}^{-}$^1^ | 0.0189576 | 0.0189383 | 0.0193216 | 0.0193239 | 0.0189722 | 0.0189630 | 0.0003743 | 0.0441681 | 0.0441682 | 0.0003366 | 0.0441682 |
| ${}_{156}^{-}$^1^ | 0.0153020 | 0.0152842 | 0.0156371 | 0.0156400 | 0.0153154 | 0.0153070 | 0.0004334 | 0.0536324 | 0.0536324 | 0.0003802 | 0.0536324 |
| ${}_{234}^{-}$^1^ | 0.0547775 | 0.0547625 | 0.0550533 | 0.0550557 | 0.0547888 | 0.0547817 | 0.0008954 | 0.0399199 | 0.0399199 | 0.0008873 | 0.0399199 |
| ${}_{235}^{-}$^1^ | 0.0344329 | 0.0344115 | 0.0348277 | 0.0348318 | 0.0344489 | 0.0344388 | 0.0003662 | 0.0431366 | 0.0431366 | 0.0003383 | 0.0431366 |
| ${}_{236}^{-}$^1^ | 0.0167107 | 0.0166943 | 0.0170200 | 0.0170220 | 0.0167231 | 0.0167153 | 0.0003839 | 0.0363782 | 0.0363782 | 0.0003437 | 0.0363782 |
| ${}_{245}^{-}$^1^ | 0.0359758 | 0.0359554 | 0.0363528 | 0.0363568 | 0.0359911 | 0.0359815 | 0.0004377 | 0.0417821 | 0.0417821 | 0.0004015 | 0.0417821 |
| ${}_{246}^{-}$^1^ | 0.0170834 | 0.0170671 | 0.0173875 | 0.0173896 | 0.0170955 | 0.0170879 | 0.0003911 | 0.0355259 | 0.0355259 | 0.0003463 | 0.0355259 |
| ${}_{256}^{-}$^1^ | 0.0138490 | 0.0138336 | 0.0141383 | 0.0141411 | 0.0138605 | 0.0138533 | 0.0004421 | 0.0473151 | 0.0473152 | 0.0003868 | 0.0473152 |
| ${}_{345}^{-}$^1^ | 0.0329816 | 0.0329645 | 0.0332966 | 0.0332997 | 0.0329943 | 0.0329863 | 0.0004844 | 0.0342896 | 0.0342897 | 0.0004434 | 0.0342897 |
| ${}_{346}^{-}$^1^ | 0.0177996 | 0.0177853 | 0.0180680 | 0.0180698 | 0.0178103 | 0.0178036 | 0.0005000 | 0.0305082 | 0.0305082 | 0.0003994 | 0.0305082 |
| ${}_{356}^{-}$^1^ | 0.0122223 | 0.0122082 | 0.0124878 | 0.0124900 | 0.0122329 | 0.0122262 | 0.0004205 | 0.0427182 | 0.0427182 | 0.0003705 | 0.0427182 |
| ${}_{456}^{-}$^1^ | 0.0120823 | 0.0120689 | 0.0123345 | 0.0123367 | 0.0120924 | 0.0120861 | 0.0004076 | 0.0393700 | 0.0393700 | 0.0003705 | 0.0393700 |
| ${}_{1234}^{-}$^1^ | 0.0387230 | 0.0387037 | 0.0390807 | 0.0390837 | 0.0387375 | 0.0387284 | 0.0004969 | 0.0511657 | 0.0511657 | 0.0004404 | 0.0511657 |
| ${}_{1235}^{-}$^1^ | 0.0277896 | 0.0277698 | 0.0281563 | 0.0281602 | 0.0278044 | 0.0277951 | 0.0002869 | 0.0491601 | 0.0491601 | 0.0002607 | 0.0491601 |
| ${}_{1236}^{-}$^1^ | 0.0163677 | 0.0163515 | 0.0166717 | 0.0166737 | 0.0163798 | 0.0163722 | 0.0003765 | 0.0439263 | 0.0439263 | 0.0003401 | 0.0439263 |
| ${}_{1245}^{-}$^1^ | 0.0277845 | 0.0277654 | 0.0281375 | 0.0281412 | 0.0277988 | 0.0277899 | 0.0003001 | 0.0475277 | 0.0475277 | 0.0002704 | 0.0475277 |
| ${}_{1246}^{-}$^1^ | 0.0154613 | 0.0154462 | 0.0157452 | 0.0157471 | 0.0154726 | 0.0154655 | 0.0003644 | 0.0410560 | 0.0410560 | 0.0003299 | 0.0410560 |
| ${}_{1256}^{-}$^1^ | 0.0124017 | 0.0123882 | 0.0126549 | 0.0126573 | 0.0124118 | 0.0124055 | 0.0004334 | 0.0448264 | 0.0448264 | 0.0003799 | 0.0448264 |
| ${}_{1345}^{-}$^1^ | 0.0269035 | 0.0268848 | 0.0272513 | 0.0272547 | 0.0269176 | 0.0269087 | 0.0002935 | 0.0472162 | 0.0472162 | 0.0002631 | 0.0472162 |
| ${}_{1346}^{-}$^1^ | 0.0157595 | 0.0157442 | 0.0160458 | 0.0160476 | 0.0157709 | 0.0157637 | 0.0003649 | 0.0412824 | 0.0412824 | 0.0003290 | 0.0412823 |
| ${}_{1356}^{-}$^1^ | 0.0125387 | 0.0125250 | 0.0127959 | 0.0127981 | 0.0125489 | 0.0125425 | 0.0004231 | 0.0440624 | 0.0440624 | 0.0003717 | 0.0440624 |
| ${}_{1456}^{-}$^1^ | 0.0117103 | 0.0116976 | 0.0119485 | 0.0119506 | 0.0117198 | 0.0117138 | 0.0004088 | 0.0411081 | 0.0411081 | 0.0003602 | 0.0411081 |
| ${}_{2345}^{-}$^1^ | 0.0281537 | 0.0281372 | 0.0284603 | 0.0284635 | 0.0281662 | 0.0281584 | 0.0003472 | 0.0450331 | 0.0450331 | 0.0003220 | 0.0450331 |
| ${}_{2346}^{-}$^1^ | 0.0140596 | 0.0140465 | 0.0143052 | 0.0143070 | 0.0140693 | 0.0140632 | 0.0003752 | 0.0373032 | 0.0373032 | 0.0003335 | 0.0373032 |
| ${}_{2356}^{-}$^1^ | 0.0108130 | 0.0108013 | 0.0110331 | 0.0110352 | 0.0108218 | 0.0108163 | 0.0004239 | 0.0406147 | 0.0406147 | 0.0003721 | 0.0406147 |
| ${}_{2456}^{-}$^1^ | 0.0107250 | 0.0107138 | 0.0109358 | 0.0109378 | 0.0107334 | 0.0107282 | 0.0004169 | 0.0385162 | 0.0385162 | 0.0003664 | 0.0385162 |
| ${}_{3456}^{-}$^1^ | 0.0098775 | 0.0098667 | 0.0100805 | 0.0100823 | 0.0098855 | 0.0098805 | 0.0003989 | 0.0372950 | 0.0372951 | 0.0003522 | 0.0372950 |
| ${}_{12345}^{-}$^1^ | 0.0207324 | 0.0207208 | 0.0209451 | 0.0209474 | 0.0207410 | 0.0207356 | 0.0002710 | 0.0219050 | 0.0219050 | 0.0002473 | 0.0219050 |
| ${}_{12346}^{-}$^1^ | 0.0121764 | 0.0121662 | 0.0123672 | 0.0123684 | 0.0121840 | 0.0121792 | 0.0003558 | 0.0200956 | 0.0200956 | 0.0003227 | 0.0200956 |
| ${}_{12356}^{-}$^1^ | 0.0098298 | 0.0098205 | 0.0100055 | 0.0100072 | 0.0098369 | 0.0098324 | 0.0004230 | 0.0235186 | 0.0235186 | 0.0003715 | 0.0235186 |
| ${}_{12456}^{-}$^1^ | 0.0091926 | 0.0091839 | 0.0093554 | 0.0093569 | 0.0091991 | 0.0091950 | 0.0004088 | 0.0219497 | 0.0219497 | 0.0003600 | 0.0219497 |
| ${}_{13456}^{-}$^1^ | 0.0092314 | 0.0092227 | 0.0093947 | 0.0093961 | 0.0092379 | 0.0092338 | 0.0003990 | 0.0208542 | 0.0208542 | 0.0003522 | 0.0208542 |
| ${}_{23456}^{-}$^1^ | 0.0079291 | 0.0079217 | 0.0080673 | 0.0080686 | 0.0079346 | 0.0079312 | 0.0003998 | 0.0186653 | 0.0186653 | 0.0003525 | 0.0186653 |
| ${}_{123456}^{-}$^1^ | 0.0073470 | 0.0073409 | 0.0074611 | 0.0074623 | 0.0073516 | 0.0073487 | 0.0003990 | 0.0149161 | 0.0149162 | 0.0003520 | 0.0149162 |
| Log-Likelihood | -1402.758 | -1402.758 | -1402.758 | -1402.758 | -1402.758 | -1402.758 | -1402.758 | -1402.758 | -1402.758 | -1402.758 | -1402.758 |
| Iterations^2^ | 10 | 10 | 11 | 10 | 10 | 10 | 15 | 5 | 4 | 17 | 5 |

lCl: lower confidence limit, uCl: upper confidence limit

^1^ ${}_{ij}^{+}$ is the dependency of the sensitivities of test i and test j; ${}_{ij}^{-}$ is the dependency of the specifities of test i and test j

^2^ The number of the iterations the whole algorithm, not the ones of the EM algorithm performed at each step

^3^Confidence limits could not be calculated
